# Personalized treatments for depressive symptoms in patients with advanced heart failure: A pragmatic randomized controlled trial

**DOI:** 10.1371/journal.pone.0244453

**Published:** 2021-01-07

**Authors:** Waguih William IsHak, Samuel Korouri, Tarneem Darwish, Brigitte Vanle, Jonathan Dang, Gabriel Edwards, Jeanne T. Black, Harriet Aronow, Asher Kimchi, Brennan Spiegel, Rebecca Hedrick, Robert Chernoff, Marcio A. Diniz, James Mirocha, Vicki Manoukian, John Harold, Michael K. Ong, Kenneth Wells, Michele Hamilton, Itai Danovitch

**Affiliations:** 1 Department of Psychiatry and Behavioral Neurosciences, Cedars-Sinai Medical Center, Los Angeles, CA, United States of America; 2 David Geffen School of Medicine at UCLA, Los Angeles, CA, United States of America; 3 Division of Health Services Research, Department of Medicine, Cedars-Sinai Medical Center, Los Angeles, CA, United States of America; 4 Cedars-Sinai Smidt Heart Institute, Los Angeles, CA, United States of America; 5 UCLA Fielding School of Public Health, Los Angeles, CA, United States of America; 6 Biostatistics Research Center, Cedars-Sinai Medical Center, Los Angeles, CA, United States of America; 7 VA Greater Los Angeles Healthcare System, Los Angeles, CA, United States of America; Brown University, UNITED STATES

## Abstract

**Objectives:**

Heart Failure is a chronic syndrome affecting over 5.7 million in the US and 26 million adults worldwide with nearly 50% experiencing depressive symptoms. The objective of the study is to compare the effects of two evidence-based treatment options for adult patients with depression and advanced heart failure, on depressive symptom severity, physical and mental health related quality of life (HRQoL), heart-failure specific quality of life, caregiver burden, morbidity, and mortality at 3, 6 and 12-months.

**Methods:**

**Trial design.** Pragmatic, randomized, comparative effectiveness trial.

**Interventions.** The treatment interventions are: (1) Behavioral Activation (BA), a patient-centered psychotherapy which emphasizes engagement in enjoyable and valued personalized activities as selected by the patient; or (2) Antidepressant Medication Management administered using the collaborative care model (MEDS).

**Participants.** Adults aged 18 and over with advanced heart failure (defined as New York Heart Association (NYHA) Class II, III, and IV) and depression (defined as a score of 10 or above on the PHQ-9 and confirmed by the MINI International Neuropsychiatric Interview for the DSM-5) selected from all patients at Cedars-Sinai Medical Center who are admitted with heart failure and all patients presenting to the outpatient programs of the Smidt Heart Institute at Cedars-Sinai Medical Center. We plan to randomize 416 patients to BA or MEDS, with an estimated 28% loss to follow-up/inability to collect follow-up data. Thus, we plan to include 150 in each group for a total of 300 participants from which data after randomization will be collected and analyzed.

**Conclusions:**

The current trial is the first to compare the impact of BA and MEDS on depressive symptoms, quality of life, caregiver burden, morbidity, and mortality in patients with depression and advanced heart failure. The trial will provide novel results that will be disseminated and implemented into a wide range of current practice settings.

**Registration:**

ClinicalTrials.Gov Identifier: NCT03688100.

## Introduction

Among patients with heart failure, depressive symptoms are prevalent and consequential [1]. The American Heart Association (AHA) and American Psychiatric Association (APA) recommend routine screening for depression among patients with cardiovascular disease in office, hospital, and rehabilitation practices, with positive screens driving assessment, referral, and treatment. For patients with depression and heart disease, both antidepressant medications and psychotherapeutic options are effective interventions for improving depressive symptoms. However, these interventions have never been compared with each other in the context of heart failure. Patients and physicians considering antidepressant medications, psychotherapy, or their combination, do not have robust evidence to inform their decisions.

The goal of the proposed research is to generate scientific evidence to help patients, caregivers, and providers make decisions about how best to manage depressive symptoms for those who have advanced heart failure (AHF). This real-world randomized pragmatic trial funded by the Patient Centered Outcomes Research Institute (PCORI) will compare the effectiveness of two evidence-based treatment approaches for treating depression: (1) Behavioral Activation (BA), a patient-centered psychotherapy that emphasizes engagement in enjoyable and valued personalized activities selected by the patient [2]; (2) Antidepressant Medication Management using the collaborative care model (MEDS).

The BA and MEDS intervention were designed to be provided via telehealth (either by telephone or by video call–depending on the patient’s preference). Telehealth delivery of treatments for heart failure patients is important because heart failure patients have a significant burden of illness that prevents them from making regular in-person clinic visits for therapy or medication supervision. Thus, telehealth delivery saves time, effort, overcomes access barriers to treatment, and improves compliance. BA and MEDS have distinct tradeoffs: BA involves a high time commitment with no risk for pharmacological side effects, whereas MEDS involves less time commitment, but carries the additional risk of side effects and potential drug interactions.

The proposed research will examine the impact of these treatment approaches on the following outcomes that patients and caregivers have identified as most important or advantageous: depressive symptom reduction, general physical and mental health-related quality of life (HRQoL), heart failure-specific HRQoL, and caregiver burden. We will also examine outcomes that patients and caregivers have identified as concerns: morbidity, as evidenced by frequent emergency department (ED) visits; hospital readmissions, longer hospital stays, and mortality. All patient/caregiver-centered outcomes will be collected at regular time intervals over the course of 12 months. The proposed study will offer clinicians specific, actionable information on how BA or MEDS, both widely used and effective interventions, perform in AHF where depressive symptoms are exceedingly common.

### Background

Depression is an important health concern, yet, it is largely under recognized and treated among patients with heart failure [3]. Approximately half of patients with heart failure experience depressive symptoms [4–6], and only half of those who are diagnosed with depression will receive treatment for depression [7]. Depression and AHF have bidirectional effects through both biological and psychosocial mechanisms [8–11]. Patients with AHF and co-morbid depression experience worse cardiac functioning [12], lower performance on stress tests [13], reduced ratings on quality of life measures [14], increases in caregiver burden [15, 16], and higher rates of ED [17, 18], and hospital use [19]. Depression severity appears to be a stronger predictor of poor HRQoL than severity of heart failure [20]. Increased severity of depressive symptoms increases risk for functional decline or death at six months among heart failure patients [21]. In a large study of outpatients with AHF, depression was found to be an independent risk factor for mortality after adjusting for confounders [22].

BA is a patient-centered and personalized psychotherapy treatment that is evidence-based as shown in more than 25 randomized clinical trials [23]. It is well-established and effective in treating depression [24], with effects comparable to cognitive behavioral therapy (CBT) and antidepressant medication [23–26]. In contrast to CBT, BA requires a less time of training for effective delivery, can be delivered by a broader range of health care workers, and has overall lower implementation barriers [23]. Thus, BA therapy is a promising psychotherapeutic approach for patients with heart failure [27], and could potentially be beneficial for their caregivers as well [28].

Antidepressants are well established for treating depression in patients with advanced medical illness [29]. Even though there is compelling evidence that antidepressants work, many patients do not receive antidepressants, largely due to the absence of a model for medication management, rather than the knowledge of which specific antidepressant is best. The collaborative care model has been used effectively to implement antidepressant medication management in AHF [30]. The impact of treating patients with AHF and co-morbid depression (using psychotherapy or pharmacotherapy) on general physical and mental HRQoL, heart failure specific HRQoL, caregiver burden, morbidity, and mortality, is largely unknown. When patients with depression are treated using the collaborative care model [31, 32], research has shown that patients respond better than usual care to antidepressants as evidenced by lower depression severity, less functional impairment and greater HRQoL at 3, 6 and 12-months follow-up [33]. The collaborative care model has been successfully implemented among depressed inpatients with acute cardiac disease [34–36].

Although the AHA recommends screening for depression, there are no formal guidelines on depressive symptom management in AHF, and there is a lack of consensus on how to best manage them. Existing antidepressant trials have not measured long-term effects and stopped at 12-week follow-up [37]. Larger and more robust randomized controlled trials (RCTs) are needed to evaluate the longitudinal effects interventions have on patients with AHF and depression [1]. There are no trials comparing BA to antidepressants or to their combination for patients with AHF and depression. There are few trials of head-to-head comparisons between pharmacological and psychotherapeutic interventions in depressive symptom treatment in cardiovascular disease [38] and none included comparing the above interventions in patients with AHF.

We present a protocol for a pragmatic, randomized, comparative effectiveness study comparing the effects of two evidence-based treatments for depression in heart failure: behavioral activation and antidepressant medication management using the collaborative care model. Furthermore, because inactivity is a symptom that is common in both depression and heart failure [7], we hypothesize that, participants receiving BA will have significantly greater improvements in depressive symptom severity, general, physical, and mental health related quality of life, heart failure specific quality of life, and caregiver burden at 3, 6 and 12 months. Furthermore, we hypothesize that those receiving BA will have significantly less morbidity and reduced mortality at 3, 6 and 12 months.

**Fig 1:**
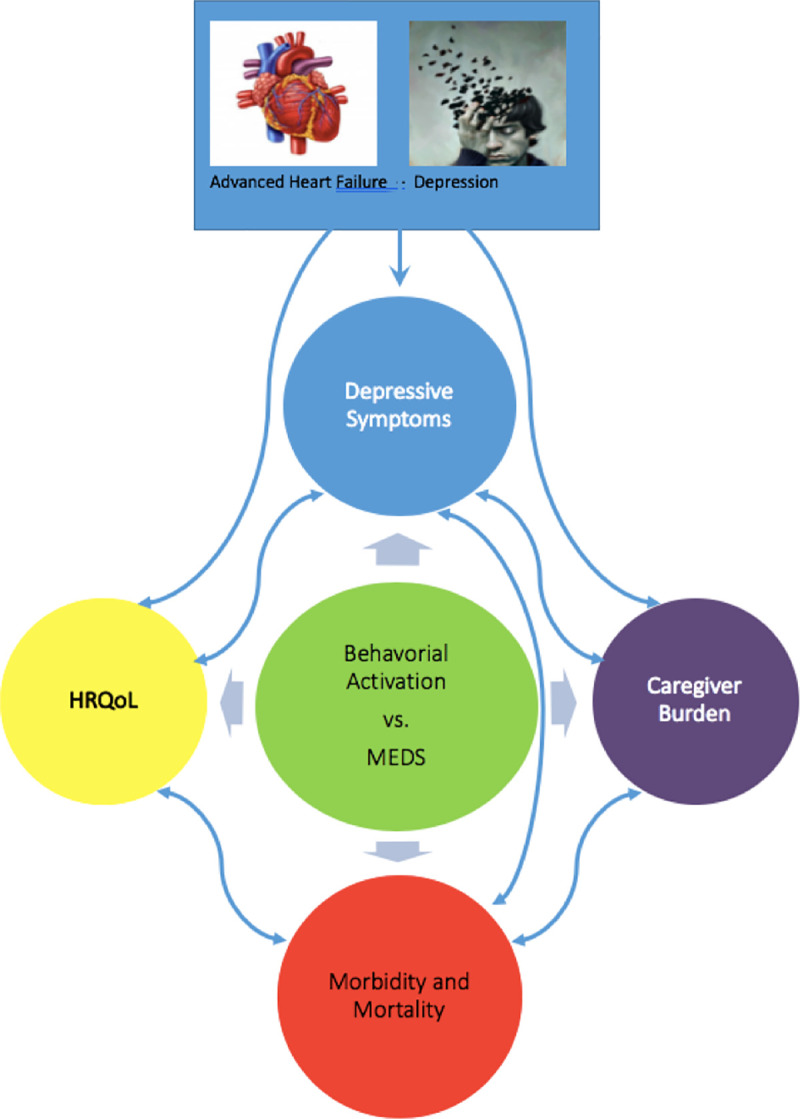
Conceptual framework.

The conceptual framework of the study is depicted below (Fig 1).

## Methods

### Study design

The Cedars-Sinai Institutional Review Board (CS-IRB) approved this study (Pro00054483). Informed consent for all research participants will be obtained via a written consent form. This is an individual, pragmatic, randomized, comparative effectiveness trial comparing two treatment arms for depressive symptom management in patients with AHF. A total of 416 patients will be randomized (in order to have 300 for analysis assuming a 28% estimated dropout rate) 1:1 using block randomization to two active treatment arms: BA and MEDS (150 patients in each treatment arm). An Intention-to-treat method will be used to preserve the notion of “as randomized, so analyzed”. As a secondary analysis, per-protocol effects will be estimated adjusting by post-randomization confounding and dose effects. Additionally, in order to ensure the study is pragmatic in its design, the study includes, as part of its leadership structure, a stakeholder-academic partnered advisory group, which consists of three consultants (two physicians who are academic experts in community participatory research, and a psychotherapy expert), patient representatives, caregivers of patients with advanced heart failure, and professionals from multidisciplinary fields related to mental health and cardiology. The stakeholder group has reviewed all main aspects of the design, the recruitment plan, intervention plans, and measures; has provided feedback to inform the design, and, in the early phases, implementation strategies and adaptations, to assure scientific and stakeholder relevance. The advisory group will continue to help monitor study progress, findings, and interpretation as well as facilitate dissemination of the findings.

### Study population

**Fig 2:**
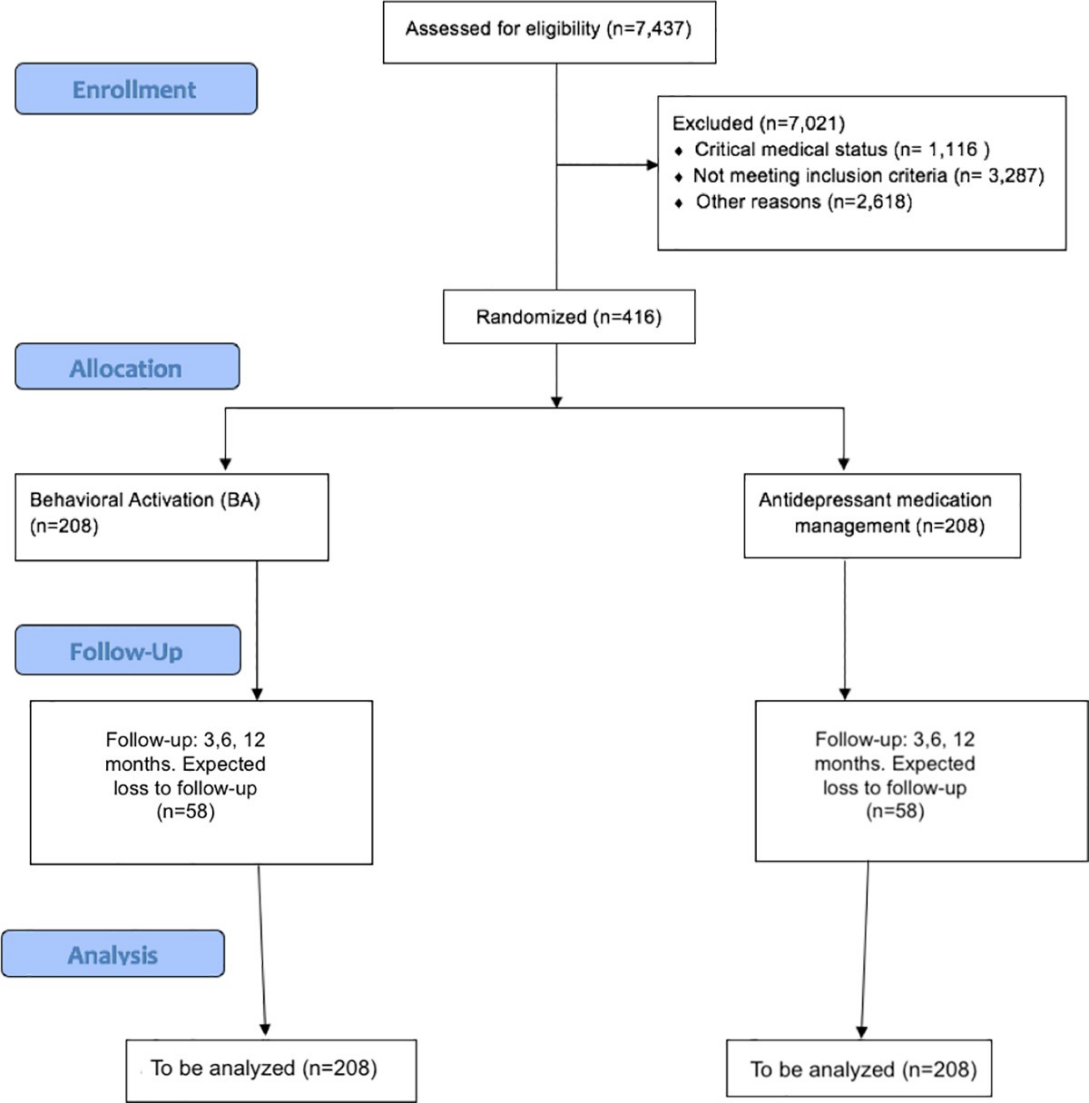
The CONSORT flow diagram.

The study population includes adults (aged 18 and over) with AHF and depression recruited from among the inpatient and outpatient populations of Cedars-Sinai, an 886-bed academic medical center located in Los Angeles. We plan to recruit a total of 416 participants to be randomized to either BA or MEDS. We estimate a 28% loss to follow-up/inability to collect follow-up data based on previous related efforts [39]. Data from 150 patients in each group after randomization will be analyzed as shown in the CONSORT flow diagram (Fig 2).

#### Inclusion criteria

Patients with AHF (as defined by New York Heart Association Classes II-IV) selected from patients admitted to the inpatient hospital at Cedars-Sinai Medical Center as well as from heart failure patients presenting to the outpatient programs of the Cedars-Sinai Smidt Heart Institute; with a life expectancy of greater than 6 months at enrollment; as well as depression as evidenced by a score of ten or greater on the nine item Patient Health Questionnaire (PHQ-9) [40] and confirmed by a diagnosis of Major Depressive Disorder, Persistent Depressive Disorder (Dysthymia), and Depressive Disorder Unspecified, as defined by the Mini International Neuropsychiatric Interview (MINI) 7.02 [41].

#### Exclusion criteria

Imminent danger to oneself or to others, cognitive impairments as evidenced by a Montreal Cognitive Assessment (MOCA) score of less than 23 [42], bipolar, psychotic, and substance induced disorders as evidenced by the MINI 7.02, and patients in active treatment of depression who are already receiving antidepressants, psychotherapy, or both, as well as an inability to communicate in English.

### Primary outcome

The primary outcome is change in depressive symptom severity at 6 months from baseline as measured by the PHQ-9.

### Secondary outcomes

The secondary outcomes measured at 3, 6 and 12 months are: general, physical and mental health related quality of life, heart failure-specific quality of life, caregiver burden, morbidity, and mortality. Measures of adherence to assigned treatment will be collected for both arms by self-report and documentation of completed visits.

### Interventions

#### Behavioral Activation psychotherapy (BA)

For patients assigned to the BA arm of the study, we will adapt the Brief Behavioral Activation Treatment for Depression (BATD), Revised Treatment Manual (BATD-R) [43]. This evidence-based, manualized treatment for depression is based on the theory that people with depression tend to stop engaging in enjoyable or meaningful activity, thus leading to a vicious downward spiral of reduced activity and worsening depressed mood. The strategy behind the treatment is to help depressed patients restore pleasurable and meaningful activity into their lives even while they are still suffering from depression. In so doing, patients can reverse the vicious cycle of anhedonia and depressed mood, enabling them to experience reductions in depressive symptoms and improvements in mood.

The original BATD-R manual was designed to be delivered over the course of ten weekly sessions. We will expand the treatment to include 12 weekly sessions, as well as three monthly follow-up sessions over three consecutive months. Ten psychotherapists will provide the BA treatment to patients. Eight of the therapists are licensed clinical social workers. Two therapists have Master of Social Work degrees but are not yet licensed. Patients will be told that the treatment will be provided remotely and that they can speak with their therapists by telephone or via a HIPAA compliant, secured video-conferencing platform (i.e., Webex). When patients have to be hospitalized due to acute health problems, their therapists will provide bedside treatment to patients in the hospital when feasible.

Therapists will be monitored and supervised by a licensed clinical psychologist and co-investigator of the study. The psychologist will provide all therapists with an initial two-hour training in the BATD-R manual as well as weekly supervision during which therapists can discuss their individual cases. Weekly supervision is intended to ensure consistent and reliable delivery of the BA treatment and to prevent therapist drift. With patient consent, the psychologist will listen in on at least one BA session provided by each therapist as a further check on fidelity to the manual. BA Therapists use the following tools to document between session goal assignment and completion as well as patient engagement in BA psychotherapy: (1) BA Patient Tracking Log where BA therapists document patients’ assigned activities from week to week, including the following week, and document the completed goals; (2) BA Survey where BA therapists keep track of patients’ engagement by having the therapists answer questions about number of sessions completed, whether the patient appears to understand, actually use and apply the treatment concepts in his/her life, and actually engage in the planned activities including new activities beyond their usual, in addition to questions about potential challenges and obstacles to engaging in BA therapy.

Therapists will introduce themselves to their patients by phone or Webex, or in person when patients are hospitalized. Patients will be provided with a booklet of worksheets and told they will use these worksheets as part of the treatment. Therapists will try contacting their patients on a weekly basis and delivering as many of the 12 sessions of the BA treatment as possible over the 12-week intervention period. The length of sessions vary depending on the individual patient, and therapists never allow sessions to exceed 50 minutes. Patients will be informed that engaging in weekly assignments between sessions is a critical component of the treatment and essential to its overall success in helping patients overcome depression.

Session 1 of the treatment provides patients with psychoeducation about depression and the rationale for why BA could potentially help them counteract depression. Patients are given the assignment of writing down how they spend their time each day on a Daily Monitoring Form and rating the enjoyment and importance of each activity on a 0–10 rating scale.

In session 2, therapists review the Daily Monitoring Forms and discuss any problems or obstacles the patients face in completing this task. Therapists use troubleshooting strategies if patients have trouble completing the forms. Therapists then introduce and discuss the Life Areas, Values and Activities Form to patients. Patients are asked to identify and explore five areas of their lives: (1) relationships, (2) education/career/volunteering, (3) recreation and interests, (4) mind/body/spirituality, and (5) daily responsibilities. Through interactive dialogue, therapists help patients articulate their values in each of the five life areas (for example, “being a loving parent” in the relationship area, or “being creative” in the recreation/interest life area). From their identified values, patients are asked to identify specific activities that represent expressions of their values in each of these areas. Patients will continue to track their current activities for the coming week on the Daily Monitoring Forms.

In sessions 3 and 4, therapists continue to help patients define and refine their values and the specific activities linked to their values which they would find enjoyable and important. Therapists help patients develop a list of 15 activities from which to choose and schedule for the coming weeks. Patients are encouraged to include activities in all five life areas, on the theory that the more activities they plan in different life areas, the more likely the odds of reducing depression. Patients are encouraged to select activities that are observable and measurable, and to break activities down into their smallest parts. Patients rank their proposed 15 activities from the least to the most difficult on the Activities Selection and Ranking Form. Patients then use the Daily Monitoring Form to plan the activities they are to engage in during the coming weeks. As each week passes, patients are encouraged to engage in their scheduled activities and to rate their level of enjoyment and importance for each activity.

Sessions 5 and 6 introduce patients to the concept that patients do not have to pursue all activities alone. They could enlist other people in their social support system to help them engage in valued activities. Patients are shown the Contracts Form and encouraged to name people who could help them engage in activities. Patients continue to report what activities they engaged in during the previous week, what obstacles and problems they faced pursuing their activities, and what activities they planned for the coming week.

In sessions 7 through 9, therapists continue to review with patients the previous week’s activities, their ratings of enjoyment and importance for each activity, and their planned activities for the coming week. In addition, therapists review each of the concepts and principles from the earlier sessions, including the concept of the five life domains, their values in each area, and the activities linked to their values. Therapists review the idea of selecting and ranking different activities, using them to plan and schedule activities for the coming week, and soliciting the help of other people to engage in valued and enjoyable activities.

In sessions 10 through 12, patients continue to report on the previous week’s scheduled activities, their enjoyment and importance, any obstacles getting in the way, and their planned activities for the coming week. With session 12 being the last weekly session, therapists explain that their next contact with the patient is going to be three consecutive monthly sessions. Patients are encouraged to continue scheduling and engaging in enjoyable and meaningful activities on their own. At each of the three follow-up sessions, patients report their activities and any problems they encounter. At the third and last follow-up session, patients are encouraged to continue engaging in activities on their own. Patients are also told that they are welcome to contact their therapist on an as-needed basis if they run into obstacles and feel that they need additional coaching to continue engaging in valued and enjoyable activities.

#### Antidepressant Medication Management (MEDS)

The MEDS intervention is delivered remotely using the evidence-based collaborative care model for depressed adults [33], using a care manager (a social worker or registered nurse) whose role is to meet with the patient in a one 50-minute introductory antidepressant medication treatment session, to educate the patient about depression and medication options and collect information on the patient/caregiver preferences/input. The care manager will then coordinate the collaboration and communication between the psychiatrist and the treating physician, resulting in starting the patient on antidepressant medication by the treating physician according the CCM guidelines [33] and study protocol antidepressant medication guidelines detailed in Table 1.

**Table 1:** Recommendations for antidepressant medications for patients with HF.

Order	Medication	Side effects (rare/overdose cases)*	Interacting Agents common to HF	Clinical Effect and Monitoring	Recommended Medication and Dose
1^st^ line	SSRIs (sertraline, paroxetine, fluoxetine)	Sexual dysfunction, GI: decreased appetite, nausea, diarrhea, constipation, dry mouth. CNS: insomnia, sedation, agitation, tremors, headache, dizziness	NSAIDS, oral anticoagulants, antiplatelets Diuretics Clonidine	Increased risk for bleeding Hyponatremia Hypothermia, sedation	Sertraline (50 mg/day)
2^nd^ line	Dopaminergic and norepinephrine agents (bupropion)	Dry mouth, GI: constipation, nausea. CNS: insomnia, dizziness, headache, agitation, anxiety. Weight loss, anorexia, myalgia, tremor, sweating, rash, hypertension	Clonidine Warfarin	Hypothermia, sedation Increased international normalized ratio	Recommended in HF patients with high BMI OR hypotension
2^nd^ line	Noradrenergic and serotonergic agents (mirtazapine)	Flulike symptoms, changes in urinary symptoms, weight gain, GI: decreased appetite, nausea, diarrhea, constipation, dry mouth, CNS: sedation, confusion. Hypotension*	Clonidine Warfarin	Hypothermia, sedation Increased international normalized ratio	Recommended in HF patients with low BMI OR Hypertension OR If patient is unresponsive to SSRIs or DNRIs
3^rd^ line	SNRI (venlafaxine, duloxetine)	Sexual dysfunction, GI: decreased appetite, nausea, diarrhea, constipation, dry mouth. CNS: insomnia, sedation, headache. Hyponatremia, hypertension	NSAIDS, oral anticoagulants, antiplatelets	Increased risk for bleeding	Use if patient was unresponsive to SSRI, DNRI or mirtazapine
X	TCAs	Orthostatic hypotension, tachycardia, QT prolongation, weight gain			Not recommended for HF patients
X	MAOIs	Orthostatic hypotension, hypertension, bradycardia, weight gain			Not recommended for HF patients
	1. Start medication according to guideline and conversation between primary care provider, patient, and caregiver (12 weeks)				
	2. If side effects occur: Consider drug-drug interactions: a. serotonin syndrome: antidepressants, opioids, stimulants, 5-HT1 agonists, herbs, mood stabilizers, antipsychotics, antiemetics (ondansetron, metoclopramide), antibiotics (linezolid) b. QTc Prolongation: SSRIs, antipsychotics, opioids, macrolides, fluoroquinolones, antiarrhythmics				
	3. Stop medication if side effects persist (10–14 days) after starting use, except sexual dysfunction				
	4. Refer to PI/Co-I in the event of: a. refractory mood symptoms b. development of psychotic or manic symptoms c. suicidal ideation *following will be reported to the Data & Safety Monitoring Board (DSMB) *PI/Co-I will be available after hours and patients will be given a 24-hour number to on-call Psychiatrist				

The care manager then contacts the patient via weekly telephone calls for 12 weeks, and then monthly for three months. The MEDS intervention is designed to be delivered by telephone because patients with heart failure have a significant burden of illness that prevents them from making regular clinic visits for antidepressant supervision. During each telephone call, the care manager will encourage patients to adhere to the antidepressant medication treatment, will track the patient’s progress or lack thereof, and will inform the treating physician and the psychiatrist regarding the patient’s condition. The psychiatrist will be available for direct consultation for patients who are not progressing, or who are experiencing adverse effects. Antidepressant medications from any class may be selected as long as the patient is maintained on the therapeutic dose according to the APA guidelines, and patient/caregiver input is always taken into account in order to personalize the MEDS intervention. After six months, patients are invited to call the care manager as needed.

**Fig 3:**
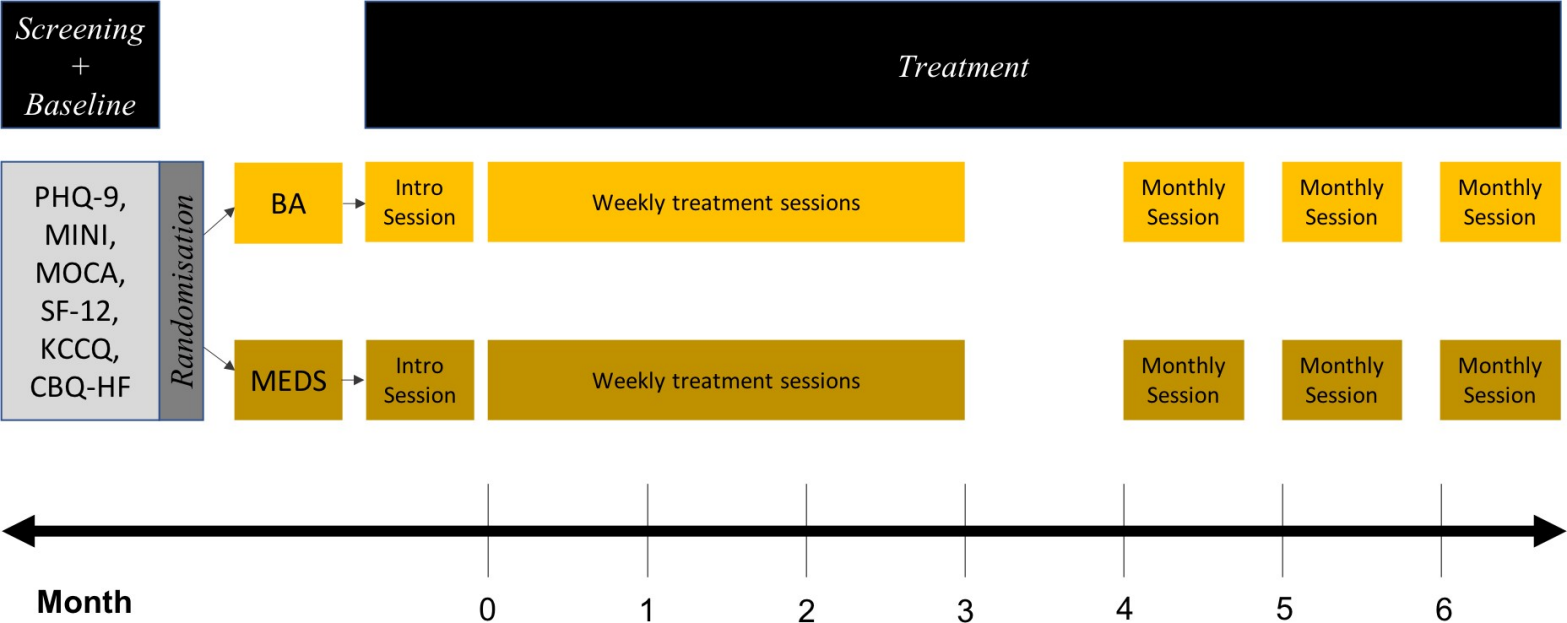
Treatment content over time. Abbreviations: PHQ-9: Nine-Item Patient Health Questionnaire, MINI: Mini International Neuropsychiatric Interview, MOCA: Montreal Cognitive Assessment, SF-12: 12-item Short Form Medical Outcomes Study, KCCQ: Kansas City Cardiomyopathy Questionnaire, CBQ-HF: Caregiver Burden Questionnaire for Heart Failure).

The treatment content for each intervention over time is shown below (Fig 3).

### Sample size

The main hypothesis to be tested is whether PHQ-9 at 6 months is different between arms (BA vs MEDS). The standard deviation for PHQ-9 was estimated equal to 5.42 based on previous literature [23] using the 95% confidence interval width asymptotic formulae for the difference between two groups, assuming equal variances. We also assumed that a minimal detectable standardized mean difference (mean difference/pooled standard deviation) for PHQ-9 between arms (BA and MEDS) is equal to 0.42 [23], which corresponds to a minimal detectable mean difference of 2.27 in PHQ-9 score units based on the estimated standard deviation. Under these assumptions, the required sample size for each group is 90 (Total = 180) patients using two-sided two sample equal-variance t-test with 80% power and a 5% significance level. We argue that 2.27 PHQ-9 score units is a clinical meaningful difference because Richards et al. (2016) [25] uses 1.9 in PHQ-9 score units as equivalence margin for their non-inferiority trial between CBT and BA. Since we expanded our recruitment to the outpatient programs, we expanded the sample size beyond the power calculation to 150 for each group (Total = 300 patients) to bring the study size close to equivalent heart failure studies and enable subgroup analyses. We estimate a dropout rate of 28%. Therefore, we plan on enrolling 416 patients (15% of to be approached eligible patients) in order to achieve a sample size of 300 patients for data analysis.

### Randomization procedure

Randomization will be performed through the REDCap (Research Electronic Data Capture) system [44]. REDCap will incorporate allocation tables generated in R [45]. Study participants will be randomized 1:1 using block randomization to the two treatment arms of the study. We will use central allocation to prevent participants, investigators, and treating clinicians from foreseeing assignment. The study design does not permit blinding of participants to the treatment received. Lack of blinding creates a possibility of a placebo effect, particularly for depression. For this reason, secondary analyses will include measures that are less likely to be subject to a placebo effect, such as hospital readmission.

### Study procedures

The research coordinator will screen potential outpatients from the Cedars-Sinai heart failure registry, as well as through the Deep-6 Cohort Builder–IRB-approved artificial intelligence software for identifying eligible patients who have visited the medical center at any point in time–and will schedule patients with a study investigator or research nurse for the baseline visit delivered either in-person or by telephone. Inpatients will be approached directly by a research nurse or study investigator. Patients will be asked if they have a caregiver who they would like to include in the study. At the baseline visit, informed consent regarding the patients’ participation in the study will be obtained. The MINI will be used to confirm depression diagnosis. The MOCA will be used to detect patients presenting with a cognitive disorder, and such patients will be excluded from participating in the study. Eligible patients will be randomized and assigned a therapist (if randomized to BA) or a care manager (if randomized to MEDS). Patient-reported outcomes will be collected at baseline, 3, 6, and 12-month timepoints using questionnaires that will be administered online using the REDCap interface [44] via the use of a smart phone/tablet, or by pencil and paper by mail and collected by a research nurse. Outcomes will continue to be collected for participants who no longer wish to participate in their assigned intervention but who still agree to receive telephone calls. Research staff will attempt to reengage participants for outcome collection to ensure completeness of data collection; only when a research participant explicitly states that they no longer want to receive any telephone calls will they be considered withdrawn and staff will cease to contact them. Patients will receive $100 for completion of the baseline visit, and $50 for each outcome collection timepoint thereafter (3, 6, and 12 months). Caregivers will receive $25 at baseline, and $10 for completion of each subsequent outcome collection timepoint.

#### Assessment of primary outcome

The primary outcome, change in depressive symptom severity, will be assessed by the 9-item patient-reported Patient Health Questionnaire (PHQ-9) at 6 months from baseline.

#### Assessment of secondary outcomes

The secondary outcome of physical and mental HRQoL will be assessed by the 12-item Short Form Medical Outcomes Study SF-12v2. Heart-Failure-Specific HRQoL will be measured by the 23-item patient-reported Kansas City Cardiomyopathy Questionnaire (KCCQ), which is the most sensitive, specific, and responsive HRQoL for heart failure [46]. Caregiver burden will be assessed by the Caregiver Burden Questionnaire for Heart Failure (CBQ-HF), a 26-item self-reported scale that measures caregiver burden on a Likert severity scale of 4 domains of physical, emotional/psychological, social and lifestyle burdens within a 4-week recall period [47]. Furthermore, we will assess morbidity by the number of ED visits, hospital readmissions, and total days in the hospital; and mortality. Hospital use will be obtained from the California Office of Statewide Health Planning and Development, and mortality data from the National Death Index, following the end of the study. Secondary outcomes will be assessed at 3, 6 and 12-months after starting treatment.

#### Additional outcomes

Adherence to assigned treatment, other treatments received for depression, and dose of anti-depressant medication will be collected by self-report. The brief medication questionnaire [48] will be used to assess antidepressant medication adherence in the MEDS arm patients after 3, 6, and 12 months of starting treatment by the research nurse. Information about the dosage of the BA intervention delivered in the BA arm patients will be collected by the research nurse from the BA therapists after 3, 6 and 12 months of starting BA treatment and will be reported as percentage of completed BA sessions. For adverse events, deaths from all causes and self-harm will be recorded and will be reported to the IRB as detailed in the Ethical Issues/Human Research section.

### Statistical analysis

#### Variables collected

The PHQ-9, SF-12, KCCQ, and CBQ-HF scores will be considered in analyses as quantitative continuous variables; morbidity, hospital readmissions, and total days in the hospital will be considered as quantitative discrete variables; and mortality will be considered as a qualitative nominal variable. The covariates that will be considered are age, sex, race, ethnicity, marital status, employment, educational level, insurance, recruitment site (Inpatient or Outpatient), ejection fraction, NYHA Class, and medical history. We also plan to account for the presence of a caregiver as a covariate when comparing PHQ-9 and other outcomes between MEDS and BA. Furthermore, we will consider medications, specifically those classified as angiotensin-converting enzyme inhibitors or angiotensin receptor blockers, β-Blockers, Loop Diuretics, Aldosterone antagonists, Opiates, and Antidepressants.

#### Analytic plan

The primary analysis will focus on PHQ-9, such that the same approach will be applied to other quality of life questionnaires. Furthermore, secondary analyses will be outlined for number of readmissions and ED visits, time to death and length of stay. All tests of hypotheses will be two-sided with a significance level of 5%. R version 3.2.5 will be used for calculations [45]. Summary statistics will be presented as percentages for categorical variables and as means ± standard deviations, medians with interquartile ranges for continuous variables. Analysis of PHQ-9 as well as other outcomes questionnaires will be performed using multivariable Generalized Additive Model for Location, Scale and Shape (GAMLSS) [49] considering baseline response, time, treatment, and interaction between time and treatment as covariables with random effects describing repeated measures at 3, 6, 12 months after treatment. The primary and secondary endpoints have their distribution support limited to positive integers. Therefore, we will compare continuous distributions in R+ starting from one-parameter distribution such as the Exponential, and we will increase the level of model complexity as needed based on residual plots up to four-parameter distributions such as the Box-Cox power exponential. While we do not expect to need to model shape and scale as functions of covariates, we will explore modeling them as functions of time. Such strategy will allow us to choose the most parsimonious distribution while obtaining an acceptable model fit.Time will be modeled as a continuous variable with random coefficients using penalized cubic splines [50] to avoid overfitting. Random effects will also be added to address the correlation among patients at the same site. In addition, covariables described above will be considered in the regression models [51]. Model diagnostics will be performed based on analysis of residual plots and worm-plots [52]. Analyses of recurring readmissions and ED visits times will be modeled with mortality using Joint Frailty Models [53] as a function of treatment to model non-ignorable missing data generated by death. Time until death will be defined as the time between index time and the event. Time between recurring events such as ED visits and readmissions will be defined as the time between the last event and the next event. Individuals who are lost to follow-up are censored at the time of the last known contact. Smooth parameters will be estimated based on Shared Frailty models. The total days in the hospital will be modeled as function of treatment using Poisson regression and variations available from GAMLSS with random effects describing repeated measures.

#### Heterogeneity (HTE) analysis

In the HTE analysis, the presence of interaction between treatment and subgroup factor (Baseline PHQ, Heart Failure Type using Ejection Fraction, Heart Failure symptom severity using NYHA Classes) will be tested in the regression model [54]. If the interaction is statistically significant, then the main analysis will be repeated for each group separately. Multiplicity will be addressed with Holm correction. As an exploratory analysis, we will consider non-parametric random effects such that patients will be clustered based on a finite mixture with their latent cluster indicators estimated using the EM algorithm. The selection of the total number of components will be pragmatic based on GAIC and diagnostic plots.

#### Per-protocol analysis

In the non-naïve per-protocol analysis, propensity scores for adherence will be estimated as a function of percentage of completed BA sessions (BA arm) and the brief medication questionnaire score (MEDS arm) at each time point and incorporated in the final models as inverse-probability weights [55].

#### Missing data

Missing data patterns for the variables with missing values will be examined using the method proposed by Little [56]. Maximum effort will be made to minimize the loss of information. If the data is found to be not missing at completely random, missing values will be imputed with values drawn from a fully conditional specification using the multivariate imputation by chained equations (MICE) algorithm [57, 58], with the number of imputed datasets chosen such that the maximum loss of efficiency of 5%. Imputed values will be checked if they are plausible as diagnostics. The outlined statistical analysis will be performed for each imputed dataset and results will be pooled according to a previously established protocol [59]. In addition, a sensitivity analysis under the assumption of missing not at random (MNAR) will be performed using pattern mixture models assuming various scenarios to assess the robustness of the results.

### Ethical issues/Human research

Participants will be informed that study participation is completely voluntary and that they can withdraw at any time. Once all data collection is complete, patient names will be replaced with a randomly assigned study identification number for the purposes of de-identification. All identifiable data will be kept on a password protected file on a stationary desktop computer and kept separate from the patient medical record and other study data. Digital files will be maintained by the onsite data manager on a secured computer. Computer entries of data will be identified by code only. Given randomization and according to PCORI standards, a Data and Safety Monitoring Board (DSMB) will be established to ensure safety monitoring of patients. The DSMB will be independent from the research team at Cedars-Sinai. Additionally, our independent research team will not modify the trial design except to ensure participant safety. The trial was also registered with ClinicalTrials.gov prior to enrolling any patients.

## Discussion

The current clinical trial details the first pragmatic, randomized, comparative effectiveness trial comparing the effects of two evidence-based treatments for depression in heart failure: behavioral activation and antidepressant medication management using the collaborative care model. Our hypothesis is that patients with AHF and co-morbid depression who receive BA will have significantly greater improvements in depressive symptom severity, general, physical, and mental health related quality of life, heart failure specific quality of life, and caregiver burden at 3, 6 and 12 months as compared to patients who receive MEDS. Furthermore, we hypothesize that those receiving BA will have significantly less morbidity and reduced mortality at 3, 6 and 12 months as compared to patients receiving the MEDS intervention. No study has compared these two treatments head to head, and thus our study can offer novel data on how depression in heart failure is best managed.

The strengths of this study lie in its power due to its large sample size and its application to the real-world due to the inclusion of a stakeholder-academic partnered advisory group as well as recruitment practices from both inpatient and outpatient sources. The utilization of BA in this study is also a strength due to BA’s low-cost and feasibility in the primary care setting as well as the behavioral approach that BA takes in targeting anhedonia and inactivity (hallmark symptoms of depression and common side effects of heart failure) as well as previous research that has shown BA to be just as effective as CBT [25], another evidence-based treatment for depression in heart failure. The long follow up time relative to other depression in heart-failure studies and the use of the evidenced-based collaborative care model presents a novel approach to testing the effectiveness of antidepressant medications in heart dailure. Furthermore, the innovative ease with which these interventions can be carried out in person, by telephone or via the use of video sessions makes the study easy to carry out and reduces the rate of patient drop-out; and is especially important for patients with heart failure who have a significant burden of illness that prevents them from making regular visits to the clinic. The use of virtual interventions allows the study to be continued during the ongoing COVID-19 pandemic, and thus will allow the study to advance to completion in the continuing pandemic conditions as well as will allow the virtual interventions to be implemented at the end of the study. Heart failure has been identified as a risk factor for severe COVID-19 [60]. Thus, the implementation of effective treatment modalities for depression in the setting of advanced heart failure, that can be administered in a socially-distant manner, take on a new urgency this year. Furthermore, it is likely the case that telehealth and virtual visits are here to stay; this study will contribute to evidence of effectiveness of such healthcare delivery modes. Finally, the proposed study interventions are straightforward to explain and easy to understand, which increases the chances of disseminating the findings successfully and increasing their visibility once implemented. The results of this study will also enhance awareness and has the potential for changing the standard of care for treating patients with AHF and depression. The limitations of the study include the patient’s burden of illness, which may prevent them from engaging in BA. Furthermore, the COVID-19 pandemic may pose a challenge to BA patients engaging in activities that they find enjoyable. Moreover, self-report BA process measures to assess goal assignment and completion were not implemented, due to significant concerns on the part of patient/patient advocates and PCORI peer reviewers regarding the burden all the existing questionnaires and additional ones would pose on patients with advanced heart failure. Baseline assessment of treatment acceptability would have enhanced the study but was not implemented due to similar concerns. Given the pragmatic design of the study, those conducting assessments are not blinded to treatment condition, thus imposing the risk of bias in participant assessments. Additionally, although the BA therapists and the MEDS care managers receive an initial two hour training on their respective study protocols as well as weekly one and half hour supervision sessions by study co-investigators, a treatment fidelity checklist and fidelity coding are not used in either condition, and thus complete adherence to protocol guidelines may not be ensured. Other limitations include the stigma surrounding depression and perceived importance of depression relative to other medical comorbidities, which may present a challenge to patient recruitment and reengagement efforts.

### Conclusions and future directions

The results of this study will be disseminated and implemented into standard practice by the study stakeholders and investigator team. If successful, this study will directly contribute to patient-centered depression care in advanced heart failure.

## Supporting information

S1 File Study protocol. (PDF)

S2 File Consort checklist. (DOC)
