# Supplementary material for: Personalized treatments for depressive symptoms in patients with advanced heart failure: A pragmatic randomized controlled trial
Source: PLoS One. 2021 Jan 7;16(1):e0244453. doi: 10.1371/journal.pone.0244453 (PMC7790529; doi:10.1371/journal.pone.0244453)
Supplement: S1 File — (PDF) [file pone.0244453.s001.pdf]

Section 1.0 General Information

Title: Personalized Treatments for Depressive Symptoms in Patients with Advanced Heart Failure
Collaborator/Sponsor: Patient-Centered Outcomes Research Institute (PCORI)
Principal Investigator: Waguih William IsHak, MD, FAPA
Co-Investigators and Key Personnel: Michelle Hamilton, MD (Co-I); Asher Kimchi, MD, FACC, FAHA, FIACS(Co-I); Itai Danovitch, MD, MBA (Co-I); Brennan Spiegel, MD (Co-I); Harriet Aronow, PhD (Co-I); Jeanne Black, PhD, MBA (Co-I); Rebecca Hedrick, MD (Co-I/Psychiatrist); Robert Chernoff, PhD (Psychologist); Marcio Diniz, PhD (Co-I/Biostatistician); James Mirocha, MS (Statistician; Gabriel Edwards, MD, MPH (Post-doc Fellow); Tarneem Darwish (Research Coordinator);Mia Mazer, CCRP (research coordinator); Research Nurses Maria Veronica Cortez, OD, BSN/RN, Desiree Triay, BSN/RN, Christine Norman, RN/B.A/B.Ed/M.Ed./PsyD., and Mia Pasini, RN/MSN.

Section 2.0 Background information

Heart Failure (HF) is a chronic syndrome affecting over 5.7 million in the US and 26 million adults worldwide. Depression is a leading cause of disability and premature mortality, affecting roughly 350 million people worldwide. Depressive symptoms in heart failure patients are common as shown by a number of studies including a Cochrane review that concluded that depressive symptoms are detected in up to 85% of HF patients. Systematic reviews place the prevalence at a range from 10-60%. Robust studies show a prevalence of 48%. Due to the heightened prevalence of depression in cardiovascular patients, the American Heart Association (AHA) recommends screening for depression among cardiovascular patients using the PHQ-2 and the PHQ-9.

Impact of HF on Individuals and Populations: Depressive symptoms are associated with poor outcomes in HF. Depression and HF have bidirectional effects through both biological and psychosocial mechanisms.

Impact on Functioning: In general, functioning impairments are closely correlated to depression severity. HF symptoms greatly restrict patients' daily physical activities. Among cardiac patients, those with HF reported more depression and significant mood disruption compared to patients with other cardiac illnesses. Among chronic illnesses, HF patients reported the poorest physical and social functioning.

Impact on HRQoL: HRQoL is markedly decreased among HF patients compared to the general population. Furthermore, a poorly rated HRQoL is more common among depressed HF compared to non-depressed HF patients and spouses. Surprisingly, the severity of heart failure is a weak predictor of HRQoL. The largest predictor of poor HRQoL was the severity of depression.

Impact on Overall Physical and Mental Health: Depressed HF patients experience worsening of cardiac functioning and perform worse on physical exams, such as the 6-minute walk test. Studies report that HF patients with more severe physical symptoms experience greater depression severity. Depressed HF patients report lower mental and physical health scores, after adjusting for relevant variables.

Impact on Caregivers: Studies have found that caregivers report feeling unprepared for the caregiving role and inadequately supported by the healthcare team, negatively impacting their psychosocial and physical health. Spouses of HF patients also report a significantly reduced well-being and feel burdened in the caregiving role. In 2004, Dracup et al., found that the spouses were at greater risk for low levels of emotional well-being and tend to feel less in control over the health outcomes of their loved one. There is also a correlation between caregiver burden and caregiver depression. Hooley et al. found that depressed caregivers (BDI-II ≥10) had much higher burden scores.

Impact on Morbidity (ED Visits, hospital readmissions, and total days in the hospital): Studies have consistently shown that ED visits are increased in depressed HF patients. Moreover, Depressed HF patients have a 4.1 times higher risk of hospitalization than non-depressed patients on antidepressants (95% Cl: 1.2-13.9 p = 0.022). A history of depression in HF patients may be a predictor of prolonged hospital length of stay. A UK study found that among 54,000 males with HF diagnosis, there were 986,000 bed-days. Among 59,000 women, there were 1.37 million bed-days.

Impact on Mortality: Increased severity of depressive symptoms increases risk for functional decline or death at six months among heart failure patients. A study of 1,017 outpatient heart failure patients, depression was found to be an independent risk factor for mortality after adjusting for confounders.

Gaps in Knowledge about the Treatment of Depressive Symptoms in Heart Failure:

Overview of the state of current research literature and trials: BA is a patient-centered and personalized psychotherapy treatment that is evidence-based as shown in more than 25 randomized clinical trials. BA is a well-established and effective treatment for depression with effects comparable to CBT and antidepressant medication. Specific to cardiac patients, BA therapy was feasible and effective, with positive impact on cardiac caregivers. Antidepressants are well established in treating depression in patients with advanced medical illness. When depressed patients are treated using the Collaborative Care model, research has shown that patients respond better to antidepressants as evidenced by lower depression severity, less functional impairment and greater HRQoL at 3, 6 and 12-months follow-up. The Collaborative Care model has been successfully implemented among depressed inpatients with cardiac disease. Although the AHA recommends screening for depression there are no official guidelines on depressive symptom management in AHF and there is a lack of consensus on how to best manage them. There are no trials comparing BA to antidepressants or to their combination for depressed AHF patients.

The gaps in evidence and the potential for the study to fill the evidence gaps:

- There are few trials of head-to-head comparisons between pharmacological and psychotherapeutic interventions in depressive symptom treatment in cardiovascular disease and none included comparing the above interventions in advanced heart failure. The proposed study aims at comparing the effectiveness of the two interventions.
- The optimal management of heart failure includes self-care and management of AHF symptoms, including depression. Self-activation or self-care have been shown to

improve patient outcomes, disease management and are associated with better patient-reported outcomes. However, psychotherapy in heart failure has been limited to CBT. The proposed study involves the utilization of BA, which has been shown to be as effective as CBT but is much more feasible yet understudied in this population.

- Existing antidepressant trials did not measure long-term effects and stopped at 12-week follow-up. Larger and more robust RCTs are needed to evaluate the longitudinal effects interventions on depressed HF patients. The proposed study extends the follow-up to one year.
- Even though there is compelling evidence that antidepressants work, many patients do not receive antidepressants, largely due to the absence of a model for medication management, rather than the knowledge of which specific antidepressant is best. The proposed study implements the evidence-based Collaborative Care Model in order to test the effectiveness of antidepressant medication management in AHF.
- The impact of treating depressed AHF patients (using psychotherapy or pharmacotherapy) on general physical and mental HRQoL, heart failure-specific HRQoL, caregiver burden, Morbidity (as evidenced by frequency of ED visits, readmissions, total days in the hospital), and Mortality, is largely unknown. The proposed study aims at examining and comparing this impact on the above outcomes at 3, 6, and 12 months.

Potential for the study to improve the quality of the evidence available to help patients and relevant stakeholders make informed health decisions and improve health care and outcomes - Potential for the study findings to be adopted into clinical practice and improve delivery of care

Improving the Quality of the Evidence: Depression is an important health concern; yet, it is largely undiagnosed and untreated in this population. Only about 50% of those who are diagnosed will receive therapy or antidepressant medication. Most antidepressant trials have focused on short-term outcomes, while the long-term effects of the patient's mood and functional outcomes have been ignored. Given the prevalence and impact of depressive symptoms in AHF, there is a need for evidence on the comparative effectiveness of psychotherapy vs. pharmacotherapy. This study will improve the quality of evidence by offering clinicians specific, actionable information on how the widely used effective interventions of BA or MEDS, perform in AHF where depressive symptoms are exceedingly common.

Adoption into Clinical Practice: In order for any study findings to be adopted into clinical practice and improve delivery of care, it would need to meet the following conditions: perceived advantage, consistency with current practices where adoption is to occur, complexity of innovation (the simpler the better), time-frame for implementation (gradual is better) and visibility once adopted (the more the better).

- The proposed study will likely be adopted if one of the two treatments (we hypothesized that it is BA) is shown to have advantage over the other in terms of the primary outcome of reduction of depressive symptom severity as well as the secondary equally important quality of life outcomes, caregiver burden, morbidity, and mortality.
- We designed our study by emulating the real-world as much as possible in order to enable its consistency with current practice, i.e., Antidepressant Medication Management using the Collaborative Care Model with a Care Manager linking the patient case with the primary care physician and a consultation psychiatrist and using technology to deliver psychotherapy to save time, effort, overcome access barriers to treatment, and improve compliance. This plan could be facilitated in future years through outsourcing to new Collaborative Care

Model online health services e.g., Concert Health, which provides remote-access using psychiatric consultants who are specialty-trained and licensed experts as a liaison and resource in providing medication consultations to PCPs.

- The innovative aspects of the proposed study are simple and user friendly, especially with the use of every day technology to receive psychotherapy and collect patient-reported outcomes. Furthermore, if BA is effective in depressed AHF patients (consistent with its effectiveness among patients with other advanced diseases) it will facilitate its use by enabling a broad range of providers to deliver the intervention[39]. We hypothesize that BA may be more efficacious than MEDS, and if this is hypothesis is confirmed by findings, then it would open the door for a feasible psychotherapeutic option which is necessary in AHF if contraindications and drug-drug effects to existing medication can be avoided.
- The implementation of our study findings will be optimal if it is done gradually in a wide variety of hospitals, primary care, and cardiology practices. Starting with depression screening, passing by identifying patient in most need of treatment, and ending with implementing the study findings using the evidence-based treatments, will likely have significant positive impact on depressed AHF patients and their caregivers.
- The proposed study contains interventions that are straightforward to explain and easy to understand making it ideal to communicate widely, thus increasing their visibility once implemented. The results of this study will also enhance awareness and lay the groundwork for an official guideline on treating depressed AHF patients.

How the research is focused on questions that affect outcomes of interest - Patient-Centeredness?

- In January 2017, we created a Health and Wellness Partnership (HWP) that meets monthly and consists of patients, caregivers, program founders such as Life Restyle Retreat, Painted Brain, Life Adjustment Team, and Clearview Treatment Programs as well as leaders from NAMI. The purpose of HWP is to actively participate in shaping research and clinical initiatives by providing patient-centered input. Two heart failure patients, who are familiar with depression struggles, joined in providing input to the proposal as it evolved from an LOI to a full application.
- Our patients and caregivers have expressed the need to focus not only on depressive symptoms but also on quality of life outcomes, which prompted us to include measures of HRQoL. They also urged us to "encourage depressed patients to get out there, and engage in fun activities, anything that may be cool, fun or different to them", which shaped our choice of the kind of evidence-based psychotherapy we chose for this study. The intervention in this study is patient-centered because BA is a personalized therapy that can be integrated into daily life, to facilitate and promote activities that are desirable and pleasurable to the individual.
- Our patients and caregivers highlighted the importance of "long-term engagement treatment plan with depressed patients", prompting us to design the study for 1-year maintenance and follow-up. Through ongoing engagement, dialogue, and debates, our patients, caregivers, and stakeholders, have identified a set of advantageous and a set of disadvantageous outcomes in depressed AHF patients for our research effort. Therefore, the proposed study aims to answer the following questions: 1. Which is the best treatment option to positively impact the most important and advantageous outcomes of improving depressive symptoms, improving general physical and mental HRQoL, improving heart failure-specific HRQoL, and reducing caregiver burden? 2. Which treatment option is most

likely to reduce the disadvantageous outcomes of Morbidity (ER visits, hospital readmissions, total days in the hospital) and Mortality?

Section 3.0 Trial objectives and purpose

Overview*: Depressive symptoms are detected in nearly half of patients with heart failure and constitute a risk factor for functional decline and death.* The goal of the proposed research is to generate scientific evidence to help patients, caregivers, and providers, make decisions about how best to manage depressive symptoms in advanced heart failure. This real-world randomized pragmatic trial will compare the effectiveness of two evidence-based treatment approaches for enhancing patient care plans: (1) Behavioral Activation (BA), a patient-centered psychotherapy which emphasizes engagement in enjoyable and valued personalized activities as selected by the patient; (2) Antidepressant Medication Management (MEDS). The proposed research will examine the impact of these treatment enhancements on the following outcomes that patients and caregivers have identified as most important or advantageous: depressive symptom reduction, general physical and mental health-related quality of life (HRQoL), heart failure-specific HRQoL, and caregiver burden. We will also examine the following outcomes that patients and caregivers have identified as disadvantageous: Morbidity (as evidenced by frequent emergency department (ED) visits, hospital readmissions, longer hospital stays), and Mortality.

All patient/caregiver-centered outcomes will be longitudinally collected in order to **assess the short, intermediate, and long-term impact** on patients' lives. Cedars-Sinai Medical Center (CSMC), a non-profit 958-bed multi-specialty academic general acute care hospital in Los Angeles, is an ideal place to perform this research as it is home to one of the largest heart programs in the US (ranked 4th in the nation) and includes the largest heart transplant center in the world; a life-saving/promoting treatment for heart failure in non-depressed patients. The Consultation-Liaison (C/L) psychiatry program is a fully staffed service with psychiatrists, psychologists, psychiatric social workers, and psychiatric nurses, where one of every ten medical inpatients is seen by C/L.

The research team has extensive experience in depression and heart failure research and its close partnership with patients, caregivers, and providers led to the development and evolution of this proposal.

Definitions:
Advanced heart failure (AHF) will be defined as New York Heart Association (NYHA) Class II, III, and IV in which patients experience shortness of breath with palpitations and physical activity limitations, characterized as moderate, severe, and at rest, respectively[1].

Depressive Symptoms will be defined as a score of 10 or above on the patient-reported 9-item Patient Health Questionnaire (PHQ) and confirmed by the MINI International Neuropsychiatric Interview for the DSM-5 (MINI 7.02) brief structured interview to meet DSM-5 criteria for current Major Depressive Disorder, Persistent Depressive Disorder (Dysthymia), and Depressive Disorder Unspecified, and not meet DSM-5 criteria for Bipolar, Psychotic, or Substance-induced Disorders.

AIMS: ***Our partnership of investigators, patients, caregivers, & stakeholders, developed the following aims:***

<u>Aim 1:</u> To compare the effectiveness of BA vs. MEDS, for depressed AHF patients.
<u>Hypothesis 1:</u> Compared to depressed AHF patients who receive MEDS, patients receiving BA will have significantly greater improvements in the primary outcome of depressive symptom severity as measured with the PHQ-9 at 6-month follow-up. Significantly greater improvements will also be detected in the secondary outcomes of general physical and mental HRQoL (SF-12v2), heart failure-specific HRQoL (KCCQ), and caregiver burden (CBQ-HF) at 3, 6, and 12 months.
<u>Aim 2:</u> To compare the impact of BA vs. MEDS on disadvantageous outcomes of Morbidity (as evidenced by ED visits, hospital readmissions, total days in the hospital), and Mortality among depressed AHF patients.
<u>Hypothesis 2:</u> Compared to depressed AHF patients who receive MEDS, those receiving BA will have significantly less Morbidity (as evidenced by less frequent ED visits, lower readmission rates, fewer total days in the hospital), and reduced Mortality at the data collection points of 3, 6, and 12 months.

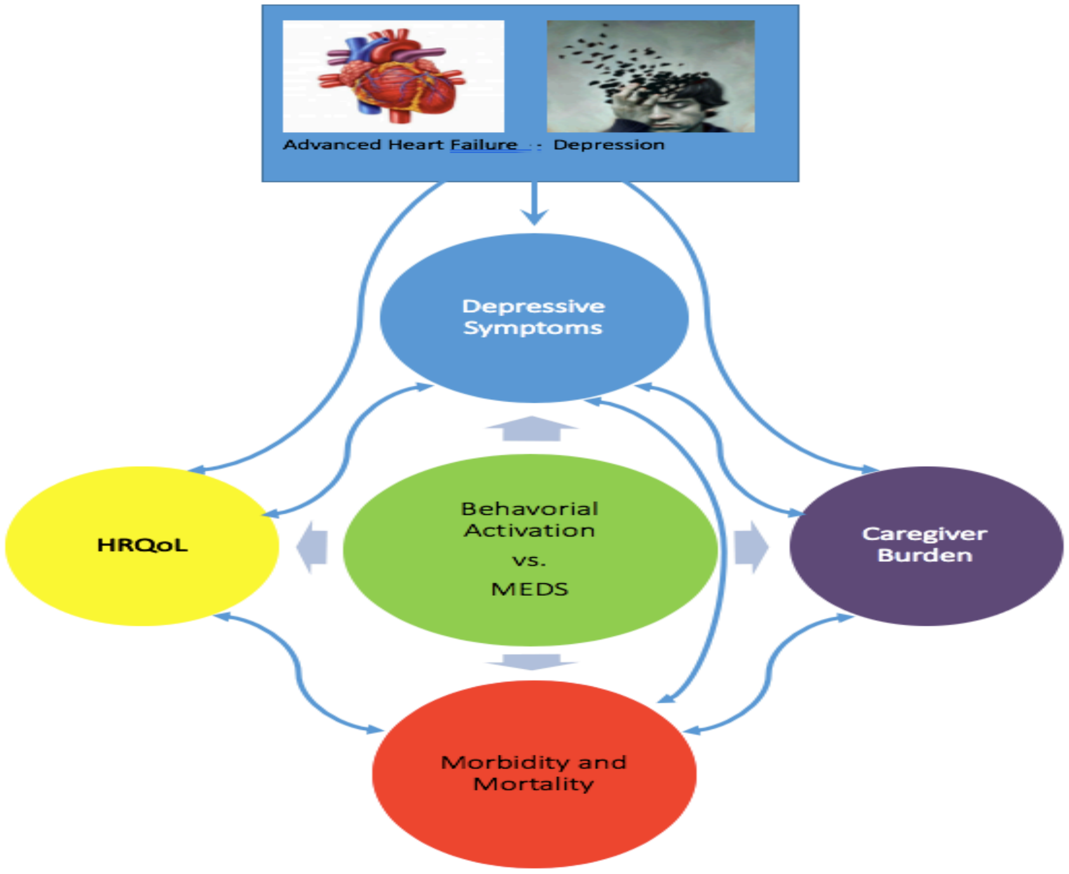

Figure 1. Conceptual Framework

Section 4.0 Trial Design

D.7.a. Overview: The design is an individual randomized pragmatic trial comparing three active treatments (BA vs. MEDS; total 300 subjects, i.e., 150 patients in each arm) in inpatients and outpatients with advanced heart failure who screen positive for depression on the patient-reported PHQ-9 (score of 10 or above) and are confirmed to be depressed using MINI International Neuropsychiatric Interview for the DSM-5 (MINI 7.02) brief structured interview to diagnose Major Depressive Disorder (MDD), Persistent Depressive Disorder (Dysthymia), and Depressive Disorder Unspecified, and to rule out Bipolar, Psychotic, and Substance-induced Disorders . The study will be initiated during an inpatient admission or an outpatient visit at Cedars-Sinai Medical Center inpatient hospital or outpatient program of the Smidt Heart Institute at Cedars-Sinai Medical Center. The study will compare the two interventions using the following patient-reported outcomes depressive symptom severity (primary outcome), general physical and mental HRQoL, heart failure-specific HRQoL, and caregiver burden at 3, 6, and 12-months after starting BA or MEDS. The study will also assess outcomes identified by patients/caregivers as disadvantageous including Morbidity (as evidenced by ED visits, readmissions, total days in the hospital), and Mortality at 3, 6, and 12 months. This Intent-to-Treat design preserves the notion of "As randomized, so analyzed". As a secondary analysis, per-protocol effects will be estimated adjusting by post-randomization confounding.

D.7.b. Study Timeline: This 3-year study has the final goal of randomizing a full sample size of 300 subjects (150 in each group). Patient recruitment and follow-up will begin in a rolling fashion after a 2-month initiation phase and will last for 20 months with an expected recruitment rate of 20-21 patients per month (10-11 patients for each group) to reach a target of 416 patients from which 28% are expected to dropout leaving 300 to analyze. The following 12 months will be spent on collecting follow-up data until reaching the last batch of patients, followed by 2 months of data analysis/manuscript preparation.

Randomization, identification and stratification of subgroups, and Data/Variables

1. Randomization: Randomization will be performed through the REDCap (Research Electronic Data Capture) system[66]. REDCap will incorporate allocation tables generated in R[82]. Study participants will be randomized 1:1 to the two treatment arms of the study.

2. Allocation concealment and Blinding: We will use central allocation to prevent participants, investigators, and treating clinicians from foreseeing assignment. The study design does not permit blinding of subjects to the treatment received. Lack of blinding creates a possibility of a placebo effect, particularly for depression. For this reason, secondary analyses will include measures that are less likely to be subject to a placebo effect, such as hospital readmission.

3. Data Collection Procedures: The surveys as detailed in the Outcome section. The variables will be extracted from the electronic medical record.

1. **Key outcomes**
    a. Primary outcome: Depressive symptom severity as measured by the PHQ at 6 months.

b. Secondary outcomes: at 3, 6, and 12 months
    i. General physical and mental quality of life as measured by the SF-12v2
    ii. Heart failure-specific quality of life as measured by the KCCQ
    iii. Caregiver burden as measured by the CBQ-HF
    iv. Morbidity as measured by ED visits, readmissions, and total days in the hospital
    v. Mortality

2. **Co-variates:**
    a. Demographics (age, sex, race, ethnicity, marital status, employment, educational level, insurance)
    b. Location of recruitment: Inpatient, Outpatient
    c. NYHA Stage: II, III, or IV
    d. Ejection fraction
    e. Baseline: PHQ-9, SF-12v2, KCCQ, CBQ-HF
    f. Medical History as detailed in the attached tables
    g. Medications as detailed in the attached tables
        i. Angiotensin-converting enzyme inhibitor or angiotensin receptor blocker
        ii. β-Blocker
        iii. Digoxin
        iv. Loop Diuretic
        v. Aldosterone antagonist
        vi. Opiate
        vii. Antidepressant
        viii. Calcium Channel Blockers
        Ix. Cannabinoid
        X. Steroids

3. **Intervention-related variables:**
    a. BA administration: Smart Phone/Table or Telephone
    b. Antidepressant medications name, dose, frequency, duration
    c. Adapted Brief Medication Questionnaire (for adherence to antidepressant medications)
    d. Percentage of completed BA sessions (for adherence to BA treatment)
    e. Antidepressant medication related side-effects (e.g. sexual dysfunction with SSRIs)
    f. Antidepressant use in the BA arm
    g. Psychotherapy use in the MEDS arm

4. **Additional Variables**

a. Caregiver information
b. Providers information: PMD, Cardiologist
c. Principal Diagnosis at enrollment

d. PHQ scores
e. Past treatment for depression
f. Past antidepressants
g. Suicide history
h. History of psychosis, anxiety, or PTSD
i. Heart failure treatment history
j. Time since HF diagnosis (years)
k. BMI
l. History of Smoking, Alcohol, or Substance use disorder
m. Allergies (Medications and Non-medications)
n. Medical co-morbidities
    i. Hypertension
    ii. Diabetes With/Without chronic complications
    iii. Obstructive sleep apnea
    iv. Atrial fibrillation or atrial flutter
    v. Myocardial infarction
    vi. Chronic obstructive pulmonary disease
    vii. Percutaneous coronary intervention
    viii. Coronary artery bypass graft
    ix. Stroke or transient ischemic attack
    x. Implantable cardiac defibrillator
    xi. Valvular disease
    xii. Peripheral vascular disease
    xiii. Hypothyroidism
    xiv. Renal failure
    xv. Liver disease
    xvi. Rheumatoid arthritis or collagen vascular disease
    xvii. Obesity
    xviii. Anemia

4. Assuring completeness of outcome data and Missing data plan: To maximize retention of study subjects throughout the follow-up period, we will do the following; First, at recruitment, we will ask patients to provide names, addresses, telephone numbers, and email addresses for 2 close relatives and/or friends, in case the patient cannot be reached. Second, we will send subjects text reminders using the IPad/Tablet (or by mail if they do not have access). Third, when we cannot reach subjects or their close contacts, we will search for address, obituaries, or US Social Security Death Index at https://www.familysearch.org/search/collection/1202535. We are implementing the Standards in the Prevention and Handling of Missing Data in Observational and Experimental PCOR (Li et al., 2012). The study staff will make every effort to ensure that data collection is complete on all the measures and variables. Having dedicated a Research Nurse assigned to each patient and collecting the data primarily using computerized method (REDCap) in addition to the study Research Coordinator as supervised by the PI will ensure completion. The primary anticipated source of missing data is patients discontinuing the study due to dropping out, illness, hospitalization, or death. If a subject discontinues

participation, the study will document the following information which will be also discussed with the DSMB: (1) the reason for discontinuation; (2) who decided the discontinuation; (3) whether the discontinuation involves some or all types of participation; (4) information on key outcomes will be collected using LOCF. Statistical methods to deal with missing data is detailed below in the analytic plan.

5. *Identifying and Assessing Participant Subgroups*: We explored a number of subgroups based on our knowledge from the literature and clinical experience. Antidepressant medication and BA psychotherapy may have different impact on depression in AHF patients, given that depressive symptom severity as measured by the PHQ-9 is an influential factor in the outcome (Bhatt et al., 2016). Depressed AHF patients with reduced ejection fraction seemed to benefit significantly more from depression interventions compared to usual care (Bekelman et al., 2018). The severity of heart failure as measured by the NYHA objective classes could have a differential benefit from antidepressants or psychotherapy in depressed patients with heart disease (Lesperance et al., 2007). Therefore, we identified three subgroups for which analyses will be performed: (a) Baseline PHQ categorized as Moderate (10-14), Moderately Severe (15-19) and Severe (20-27) with the exploratory hypothesis that BA will be superior to MEDS in the Moderate and Moderately Severe groups, but not for the Severe group; (b) Heart Failure Type defined as reduced ejection fraction ≤40% and >40% with the confirmatory hypothesis that BA is superior to MEDS in both groups, and (c) Heart Failure symptom severity using NYHA Objective Classes (Class II, III and IV) with the exploratory hypothesis that BA is superior to MEDS in class III and IV, but not for class II.

Overview of Study Protocol/Procedures

1. The study will recruit from heart failure patients admitted to the inpatient hospital at Cedars-Sinai Medical Center as well as from heart failure patients presenting to the outpatient program of the Smidt Heart Institute at Cedars-Sinai Medical Center. At both sites, when a patient is identified to have the diagnosis of Heart Failure, the inpatient or outpatient nurse will screen the patient for depressive symptoms using the PHQ-2. If the patient is answers positively to one of the two questions, PHQ-9 will be administered. If the score is ≥10, the nurse will notify the treating physician. The treating physician will ask the patient if they have any interest in taking part in the proposed study and/or would like to learn more about it. If the patient agrees, a Co-Investigator (Co-I) will be assigned to the patient to educate the patient and the caregiver about depression and treatment options including alternatives, risks, and potential benefits. The patient and the caregiver will be informed about the study and will be given a blank copy of the informed consent with the patient to read and consult with loved ones if necessary. A blank caregiver informed consent form for the data collection about the caregiver burden will be also given.

2. The PI or Co-I/designee will meet with the patient for informed consent. If the patient consents to the study, the patient will be given a copy of the signed consent. Data on the caregiver burden will be collected only after the caregiver signs their informed consent form to participate. If the patient does not agree to the study, they would continue treatment without undue influence of their decision on their medical care. The meeting will be held either in-person or via an approved e-consenting procedure.

3. Co-I/designee will administer the MINI International Neuropsychiatric Interview for the DSM-5 (MINI 7.02) brief structured interview to diagnose Major Depressive Disorder, Persistent

Depressive Disorder (Dysthymia), and Depressive Disorder Unspecified, and to rule out Bipolar, Psychotic, and Substance-induced Disorders.
4. Co-I/designee will check whether the patient meets the inclusion and exclusion criteria detailed in section D.10.a. This will include using the Montreal Cognitive Assessment (MoCA)[64], to exclude depressed AHF patients with significant cognitive impairments if the MOCA score is < 23[65] as well as confirming with the treating physician that the patient's condition is not terminal, i.e., life expectancy is > 6months.
5. If the patient meets criteria, the patient will be enrolled in the study and assigned a *Research Nurse*.
6. The Research Nurse will collect all the variables from the patient, caregiver, and the medical record including information on baseline co-morbidities.
7. The Research Nurse or Designee will then randomize the patient into BA or MEDS.
8. The Research Nurse will train the patient and the caregiver on the use of their own Smart Phone/iPad/Tablet to collect outcome measures. The patient will take the study Patient-Reported Measures (PHQ-9, SF-12v2, and KCCQ) at baseline on their own Smart Phone/iPad/Tablet using the REDCap interface[66]. If the patient does not have access to or is unable to use a Smart Phone/iPad/Tablet, paper and pencil forms will be used. The Research Nurse will authorize an incentive payment of $100 for Baseline Measures and $25 for the caregiver burden measure.
9. If the patient is randomized to the BA arm, the Research Nurse or designee will be assign the patient a *BA Therapist* who will provide BA following the BA guidelines described below, starting with an introductory treatment session, preferably in the presence of the patient's caregiver, delivered using their own Smart Phone/iPad/Tablet in order to work through any future user problems, followed by 12-weekly 50-minute sessions using their own Smart Phone/iPad/Tablet. A visit is considered missed, if it doesn't occur once a week (anytime within that week is acceptable as it is based off the patient's schedule). If the patient does not have access to or is unable to use a Smart Phone/iPad/Tablet, a telephone will be used. After 3 months, BA sessions will change to monthly sessions for three months. Contact thereafter will be on as needed basis.
10. If the patient is randomized to the MEDS arm, the Research Nurse or designee will be assign the patient a *Care Manager* who in collaboration with the treating team, and taking into account the patient/caregiver input, will coordinate starting the patient on antidepressant medication following the Collaborative Care Model[46] (CCM) guidelines described below. The role of the Care Manager is to oversee the antidepressant medication management care that the patient will receive, and to liaison between the patient, the treating physician, and the psychiatrist involved in the patient's care. Antidepressant medications will be maintained throughout the course of the study. The Care Manager will maintain contact with the patient/caregiver on a weekly basis in the first 12 weeks, then on a monthly basis for 3 months, and then on as needed basis thereafter. For the 12 weekly visits, A visit is considered missed, if it doesn't occur once a week (anytime within that week is acceptable as it is based off the patient's schedule).
11. After starting treatment, the integrity of the delivery of BA or MEDS according to the research protocol guidelines will be supervised and monitored by study Co-I who will on a quarterly basis: (1) review each patients' medical records and contact the BA Therapists and Care Managers, to ensure the integrity and adherence to the research protocol guidelines.
12. At the Patient-reported outcome measurement points of 3, 6, and 12 months from starting treatment, the Research Nurse will contact the patient to take the Patient-Reported Follow-up

Measures on their own Smart Phone/iPad/Tablet or by Mail and authorize incentive payment of $50 each time for Follow-up Measures and $10 each time for the caregiver burden measure.
13. At the Morbidity and Mortality outcome measurement points of 3, 6, and 12 months from starting treatment, the Co-I/Designee will collect the data on Morbidity (ED visits, readmissions, total days in the hospital), and Mortality.
14. All medications (dose, frequency, and duration), medication side effects, medication-interactions will be collected and recorded by the Research Nurse.
15. All treatment discontinuations and protocol deviations will be recorded in detail by the study Research Coordinator. Whereas participant drop-out and added interventions are important in any community-based RCT, in a pragmatic study these factors are extremely salient to the understanding of the comparison between effective interventions. Adherence to the assigned intervention will be measured directly by tracking missed "visits" and monitoring "missed/inaccurate medication use". Furthermore, we will report information about the number of BA patients who are subsequently prescribed antidepressant medications and MEDS patients who actively enroll in psychotherapy.

Summary of the Timeline, Sample Size, and Recruitment Plan: The study will run for three years and aims at analyzing data on 300 subjects (150 in each group). Given a 28% dropout rate, the study will recruit 416 patients (208 patients per arm). After 2 months of initiation phase including staff training, the study will recruit patients in a rolling fashion for 20 months. The expected recruitment rate is 20-21 patients per month (10-11 patients for each group). The study will collect follow-up data after 12 months, then 2 months will be spent on data analysis and manuscript preparation.

Section 5.0. Selection and Withdrawal of Subjects

The study population will be selected from all patients at Cedars-Sinai Medical Center who are admitted with heart failure and all patients presenting to the outpatient program of the Smidt Heart Institute at Cedars-Sinai Medical Center. The study population will consist of AHF patients (as evidenced by NYHA class II, III, or IV), with life expectancy > 6months (as evidenced by confirmation with the treating physician), who are depressed (as evidenced by a PHQ-9 score of ≥10, with a confirmed DSM-5 Depressive Disorder diagnosis (as evidenced by the MINI 7.02), with no imminent danger to self or others (as evidenced by , not experiencing cognitive impairments (as evidenced by a MOCA score of ≥23) or Bipolar/Psychotic/Substance-induced Disorders (as evidenced by the MINI 7.02), and are not being actively treated for depression (as evidenced by not receiving antidepressants, psychotherapy, or both). The inclusion and exclusion criteria are detailed in Table 1.

**Table. Inclusion Criteria**	**Exclusion Criteria**
1. HF New York Heart Association classes: II-IV.<br>2. Life expectancy of more than 6 months.<br>3. PHQ-9 score ≥10.<br>4. Diagnosis of Major Depressive Disorder, Persistent Depressive Disorder (Dysthymia), and Depressive Disorder Unspecified, as confirmed by the MINI 7.02.	1. Imminent danger to self or others.<br>2. Cognitive impairments with a MOCA score of < 23.<br>3. Bipolar, Psychotic, and Substance-induced Disorders.<br>4. Patients in active treatment of depression who are already on antidepressants, psychotherapy, or both.

Recruitment and Enrollment: We will recruit patients who present to the inpatient and outpatient heart failure programs at Cedars-Sinai Medical Center (CSMC) who have a diagnosis of heart failure (Class II-IV) and screen positive for depression using a score ≥10 on the Patient Health Questionnaire (PHQ) for depression. We intend to use an additional inpatient IRB approved and HIPAA compliant recruitment tool called the 'Deep 6 Cohort Builder' a software application designed to efficiently and effectively, identify and recruit eligible patients for clinical trials. Additionally, patients will be recruited from psychiatry consults ordered on AHF patients with depression. The study will be promoted via printed flyers, which will be posted on CSMC campus and physician newsletters. Co-Is will be responsible for informational sessions about the study protocol with cardiologists and internists on medical staff at Cedars-Sinai as well as those in the community as they have done before during the $10 million BEAT-HF study at CSMC.

**Table. Recruitment**	**Patients**	**Caregivers**
Estimated number of potentially eligible study participants: The Cedar-Sinai inpatient heart failure program treats 1,750 patients per year (latest figure from 2017) yielding 3,062 potentially eligible study participants in the course of 21 months of enrollment. Additionally, the outpatient program treats 2,500 patients per year, yielding another 4,375 potentially eligible study participants in the course of 21 months of enrollment.	7,437	7,437
Total number of study participants expected to be screened: 15% of participants will be excluded from screening due to critical medical status (Thoma, 2010).	6,321	6,321
Total number of study participants expected to be eligible of those screened: 48% (3,034) are expected to screen positive for depression[8].	3,034	3,034
Target sample size: We plan to enroll 416 study participants (15% of patients approached) with an estimated maximum dropout rate of 28%, in order to have 300 study participants (150 in each arm) for final data analysis.	416	416
If applicable, total number of practices or centers that will enroll participants:	2	
Projected month first participant enrolled (month after project initiation):	11/2018	
Projected month last participant enrolled (month after project initiation):	6/2020	
Projected rate of enrollment (anticipated number enrolled per month of enrollment period):	20-21/month	
Estimated percentage of participant dropout:	28%	

Retention: We focused on improving our retention plan, and estimate a dropout rate of 28% using the following interventions especially the ones that our research team that conducted the BEAT-HF study had mastered:

1. If the patient meets inclusion criteria, the patient will be enrolled in the study and assigned a Clinical Research Nurse (CR-RN) who will perform their role according to the model described above. Retention in this study is largely connected to relationship building with the CR-RN who is expected to be available, reachable, resourceful, and will maintain frequent contact with all patients according to the study procedures described above.

2. We will adopt a risk identification system with pre-planned retention strategies as detailed by Zweben, Fucito, and O'Malley in 2009, and summarized in the table below (Zweben, 2009).
3. We will consider the implementation of evidence-based models such as AASAP (anticipate, acknowledge, standardize, accept, plan), to improve retention of patients (Fisher, 2012).
4. We will take more of an engagement approach toward consenting patients rather than mere retention, as described in the engagement efforts by the Clinical Trials Transformation Initiative's (CTTI) (Patrick-Lake, 2018).

This study does not propose any exclusion criteria based upon gender or minority status. Subjects from both genders and in all ethnic and racial categories will be actively recruited. The ethnic/racial distribution in the Cedars-Sinai Medical Center demographic catchment area is approximately 10% African-American, 10% Asian, 40% White/non-Hispanic, and 40% Hispanic. We expect a recruitment pool in roughly the same percentages. All of our recruitment efforts will clearly state that we encourage women, minorities and those from disadvantaged backgrounds to contact the research team for more information about participating in research. Although depression is more common among females, we intend to split enrollment equally between the genders. Balancing gender (50% / 50%) in the sample size maximizes the chance of detecting a gender difference if one is present, with the smallest number of cases.

Inclusion of Children: Because some medical inpatients are children ages 18-21, we may enroll them in our study. By definition (CFR46.102), "children are persons who have not attained the legal age for consent to treatments or procedures involved in the research, under the applicable law of the jurisdiction in which the research will be conducted." However, both the State of California and our local IRB allow subjects over 18 years of age to provide legal consent to participate in research. Individuals under the age of 18 are being excluded from this research because the purpose of the project is to validate a measure of depressive symptoms designed for an adult population, developed on individuals aged 18 years or older.

Estimated Final Racial/Ethnic and Gender Enrollment Table: We plan on enrolling 416 patients (dropout rate of 28%) in order to achieve a sample size of 300 patients for data analysis.

	Ethnicity				Total (N)
Race	Not Hispanic or Latino		Hispanic or Latino		
	Male (N)	Female (N)	Male (N)	Female (N)	
American Indian/Alaska Native	3	2			5
Asian	20	21			41
Hawaiian/Pacific Islander	3	3			6
Black or African American	16	15	3	3	37
White	124	124	25	25	298
More than one race	14	15			29
**Total (N)**	180	180	28	28	416

Section 6.0 Treatment of Subjects

There is currently no standard of care for treating depression in patients with heart failure.
Behavioral Activation Psychotherapy (BA):

Evidence of Effectiveness and Widespread Use: BA is an evidence-based psychotherapy with more than 25 randomized clinical trials showing effectiveness in depression. Past meta-analyses and reviews established BA as an effective treatment and viable alternative to other psychotherapies. BA effects are comparable to CBT and antidepressant medication. BA was shown to be both feasible and effective in cardiac patients (Busch, 2017). Moreover, BA had a positive impact on cardiac caregivers, and it may be a non-pharmaceutical treatment of choice in cardiac patients.

Intervention: The patient is introduced to BA using a one 50-minute Introductory BA Treatment Session, followed by 12 weekly 50-minute Outpatient Treatment Sessions, then 3 monthly 50-minute Outpatient Maintenance Sessions, preferably attended by the patient's identified caregiver. Contact thereafter will be on as needed basis. A typical BA session will last 50 minutes and include a review of the previous session and completed daily monitoring record forms, an in-depth discussion of life areas and value, and verbal reinforcement of activity engagement. The patient assignment will be to chart progress of activity engagement by duration, frequency, self-reported level of mastery, review of engagement in 3-5 activities considered rewarding and important and will provide support and encouragement. Based on input from our patient/caregiver group, the BA therapist will provide encouragement to participate in an in-person or online "Meetup" /similar group for the activity of the patient's choice. All sessions will be delivered via Telemedicine (Smart Phone/iPad/Tablet), or telephone calls in case of lack of access or inability to use the equipment. The therapist will be a licensed mental health practitioner of any level except doctoral level to enable implementation and dissemination in a variety of settings. The BA sessions will be in accordance of the revised treatment manual for depression[69]. The therapists will receive a 2-hour training on the BA application study protocol, as well as BA theory, technique, and evidence and will also receive weekly 1.5-hour supervision to ensure adherence to study protocol (all by BA expert).

Antidepressant Medication Management (MEDS):

Evidence of Effectiveness and Widespread Use: Evidence has been well established for the effectiveness of antidepressants in patients with advanced medical illness[43]. MEDS delivered using the Collaborative Care Model (CCM) has been shown to lead to lower depression severity, less functional impairment and greater HRQoL at 3, 6 and 12-months follow-up. CCM is a real-world, flexible, and personalized treatment approach that involves active engagement of the patient, treating physician, and psychiatrist, highly coordinated by a non-physician Care Manager. MEDS using CCM has been successfully implemented among depressed inpatients with cardiac disease and has been shown to enhance patient care after discharge from inpatient to outpatient settings, which commonly incorporates telemedicine, with high effectiveness.

Intervention: The MEDS intervention will be delivered using the evidence-based Collaborative Care Model (CCM) for depressed adults using a Care Manager. A Care Manager can be a nurse, nurse practitioner, physician's assistant, or social worker. After randomization to MEDS, the Care Manager will meet with the patient in a one 50-minute Introductory Antidepressant Medication Treatment Session, to educate the patient about depression and medication options and collect information on the patient/caregiver preferences/input. The meeting will be either

conducted in person or through approved online meeting The Care Manager will then coordinate the collaboration and communication between the psychiatrist and the treating physician, resulting in starting the patient on antidepressant medication by the treating physician according the CCM rules and study protocol MEDS guidelines. The Care Manager will maintain contact with the patient/caregiver on a weekly basis in the first 12 weeks, then on a monthly basis for 3 months, and then on as needed basis thereafter. The Care Manager will communicate questions, problems, medication changes between the patient, the treating physician and the psychiatrist. The Care Manager will encourage patients to adhere to antidepressant medication treatment, will inform the treating physician and the psychiatrist of the patient's progress or lack of thereof, and will maintain contact with the psychiatrist to review progress of the patient's treatment plan and the psychiatrist will be available for direct consultations for patients who are not progressing. For the purpose of this study, MEDS will be initiated or continued according to the following requirements: Antidepressant medications from any class could be selected as long as the patient is maintained on the therapeutic dose according to the APA guidelines, and patient/caregiver input is always taken into account, in order to personalize MEDS. Handouts/ and brochures will be provided to the patient and the patient's primary care provider including guidelines for choosing antidepressants in depressed AHF patients. These guidelines include drug-drug interactions with heart-related and other medications that the patient may be prescribed**.** The Care Managers will receive a 2-hour training on the MEDS application study protocol, as well as CCM technique, and evidence and will also receive weekly 1.5-hour supervision to ensure adherence to study protocol by Co-I.

Section 7.0 Assessment of Efficacy

This is a comparative effectiveness pragmatic trial, i.e., it is not a trial to establish efficacy of an intervention, but to determine which intervention is more effective in the real world.

Overview: Patient-reported outcomes will be collected at pre-determined timepoints using questionnaires that will be administered online using the REDCap interface through the use of a smart phone/tablet, or by pencil and paper by mail or collected by the Research Nurse. The primary outcome measure will be patient self-reported depressive symptom severity using PHQ-9 at 6 months from the start of treatment. The secondary outcomes will be self-reported general physical and mental HRQoL (SF-12v2), heart failure-specific HRQoL (KCCQ), caregiver burden measure, in addition to Morbidity (as evidenced by ED visits, readmissions, and total days in the hospital), and Mortality rate, at baseline and after 3, 6, and 12-months from the start of treatment. The following outcome measures will be analyzed in all study participants:
Depressive Symptom Severity: The 9-item patient-reported Patient Health Questionnaire (PHQ-9).
Physical and Mental HRQoL: The 12-item Short Form Medical Outcomes Study SF-12v2 for assessment of Physical and Mental HRQoL.
Heart Failure-Specific HRQoL: The 23-item patient-reported Kansas City Cardiomyopathy Questionnaire (KCCQ) which is the most sensitive, specific and responsive to HRQoL for heart failure.
Caregiver Burden: The Caregiver Burden Questionnaire for Heart failure (CBQ-HF) is a 26-item self-reported scale that measures caregiver burden on a Likert severity scale of 4 domains of physical, emotional/psychological, social and lifestyle burdens within a 4-week recall period.

<u>Patient/caregiver disadvantageous outcomes of Morbidity (as evidenced by ED visits, hospital readmissions, total days in the hospital), and Mortality</u> each interval of 3, 6, and 12-months after starting treatment.
<u>Additional outcomes:</u> Adapted Brief Medication Questionnaire will be used to assess adherence to the intervention in patients after 3, 6, and 12 months of starting treatment by the Research Nurse. Information about adherence to BA in the BA arm patients will be collected by the Research Nurse from the BA therapists after 3, 6 and 12 months of starting BA treatment and will reported as percentage of completed BA sessions. For adverse events, all deaths from all causes and self-harm will be recorded and will be reported to the IRB as detailed in the Human Protection section.

Section 8.0 Assessment of Safety

Protection of Human Subjects
This Human Subjects Research meets the criteria for Section 5, titled "Human Subjects Research Policy, from the "Supplemental Grant Application Instructions for All Competing Applications and Progress Reports." *(a component of the PCORI funding application)*

<u>Risks to the Subjects</u>
<u>1. Risks to Human Subjects Involvement, Characteristic and Design:</u> The study is a randomized controlled trial (RCT) that will enroll 300 patients with heart failure who screened positive for depression using the PHQ-9 score of 10 or above. The study aims to compare the effectiveness of Behavioral Activation (BA) vs. antidepressant medication management (MEDS). We will randomize patients using the MS Excel random number generator to ensure that there is an equal distribution of participants in the study groups. We will measure changes in self-reported depressive symptom severity, general physical and mental HRQoL, heart-failure-specific HRQoL, and caregiver burden. We will also measure Morbidity (as evidenced by ER visits, readmissions, and total days in spent in the hospital) as well as Mortality. Research material will be collected specifically for this research project and will include interview data, patient-rated and clinician scales, and physical and laboratory examinations. All subjects will be assigned a unique code number to protect personal privacy. All data and samples will be stored according to code number and the only personal identifiers that will be associated with a sample are sex, age, and ethnicity. The code (with linkages) and any identifying data will be kept in a password-protected file located in a room separate from the other study files. The hard copy data files (documentation obtained from interviews, diagnostic procedures and testing) will be stored in the CSMC Psychiatry Research Offices. Digital files will be maintained by the onsite data manager on a secured computer. Computer entries of data will be identified by code only.

<u>Data Management:</u> Data will be collected and recorded into a database system. Each record will include the subject's unique identifying number, demographic information, visit type, visit date, total scores and individual item scores on each of the measures. The Data Management Coordinator (study Research Coordinator) will review data integrity in terms of accuracy (using error alerts) and completion of all items on a daily basis. A record of the data will be printed for each visit and will be kept under double lock and key at the research data storage bop in the Psychiatric Research Division. An electronic backup copy of the data will be saved on the secure

server upon data entry, in addition to weekly CD disks. A separate data copy on CD will be stored on a weekly basis offsite under double lock and key to enable data retrieval in the event of a catastrophe preventing access to the facility or the data. A backup CD will be created on a quarterly basis. The PI will oversee compliance with the procedures set forth. The database will be designed and managed by the Cedars-Sinai Biostatistics and Bioinformatics Research Center (BBRC)– a group that provides support for clinical trials throughout CSMC. Reports generated from the database will provide a basis for ongoing monitoring of subject accrual and retention, as well as completeness of data. These will be used to identify and resolve problems that may arise. Back-up copies of the database will be created daily per CSMC EIS standard protocol for all hospital-wide data servers. Access to data will be restricted to the study team but the code linking subject identification # to patient health information (PHI) will be restricted to only the PI and the study Research Coordinator. Data will only be released to a third party in an emergency medical situation or when required by law.

2. *Risk due to Study Procedures:* Minimal psychological or emotional discomfort may arise due to the screening procedure and interviews /questionnaires conducted at each study visit. The instruments administered during the course of the study pose no specific risks or discomforts beyond those of a standard clinical interview situation such as feeling upset at a review of the psychiatric status or experiencing boredom or fatigue. There are questions that will be asked that may cause some subjects to feel uncomfortable. However, it will always be emphasized that all questions are optional, and participation is voluntary. Each subject will be screened for suicidal ideation and intent prior to study entry and any subject who is actively suicidal will be excluded from study participation and referred for appropriate care. The subject will be reminded that he/she may always decline to participate in this study and he/she may discontinue at any time.

3. Potential Risks: Behavioral activation will have minimal risk to the patients as study participation consists of education, learning, and completing a number of self-administered questionnaires and extracting data from a patient’s medical chart. Patients using antidepressant may experience side effects from the medication, including bleeding risk, bradycardia, hypertension, electrocardiogram (EKG) changes, reduced cardiac conduction and output, arrhythmias, and sudden death. If patients experience these symptoms, they will be asked to stop using the antidepressant. To minimize the side effects, we will start with a low dose and work our way to the therapeutic dose with closed-monitoring for adverse effects mentioned above.

4. Adequacy of Protection against Risks: All patients meeting the eligibility criteria will be enrolled in the trial. The patient will receive an Informed Consent detailing the protocol, including research procedures and requirements for follow-up, as well as the risks and benefits of the study participation. The PI, Research Coordinator, and the research support team contact information will appear on the Informed Consent. Subjects will be informed that study participation is completely voluntary and that they can withdraw at any time. Once all data collection is complete, patient names will be replaced with a randomly-assigned study identification number for the purposes of de-identification. All identifiable data will be kept on a password protected file on a stationary desktop computer and kept separate from the patient medical record and other study data. The computer contains firewall and real-time anti-virus and anti-spyware protection that is kept up-to-date.

B. Vulnerable Populations: The study does not involve pregnant women, human fetuses/neonates, or prisoners.

C. Data and Safety Monitoring Plan (DSMB): Given randomization and according to PCORI standards, a DSMB will be enacted to ensure safety monitoring of patients. The DSMB will be independent from the research team at CSMC. As specified by PCORI, the DSMB will have an appropriate Chair and staff to monitor study safety, minimize research-associated risk, review and report adverse events to the IRB. DSMB will be composed of senior investigators with experience with intervention studies in the community as well as studies of depression. The primary goals of the DSMB are as follows: 1) To monitor and advise on scientific and ethical issues related to the study implementation for the protection of human subjects, 2) To review and approve the protocol and subsequently conduct annual reviews to determine whether participant safety has been adequately safeguarded, 3) To review procedures and decisions regarding the adequate protection of specific participants when investigators break protocol because of adverse events or clinical deterioration, 4) To review progress to see that enrollment goals have been met, 5) To monitor and advise on ethical issues related to adverse events, 6) To oversee the confidentiality of data, and quality of data collection, management, and analysis, 7) To recommend, if necessary, discontinuation, modification, or termination of the study based upon emerging data (in the study and literature) and evaluation of risk/benefit ratio, and 8) When possible, to serve as the final arbiters of whether individuals should be removed from the study. Although our treating clinicians are empowered to take whatever immediate action is necessary to safeguard the welfare of individuals, the DSMB will be called upon whenever possible to render judgments in the advent of serious suicidal intent or clinical deterioration. The DSMB will meet once per year, as well as needed. In advent of emergencies, the DSMB will meet via teleconference. For each meeting, the DSMB will first meet in an open session attended by the PI. The group will first review the research protocol and plans for data and safety monitoring. This group will be used to review any problems in implementing the safety plan and for suggesting any necessary modifications to the safety plan. The DSMB will then meet in a closed session for the purpose of reviewing emerging trial data. Confidentiality will be maintained by providing data without any identifying information to the committee. At the conclusion of the meeting, the DSMB will make recommendations to the PI and the Internal Review Board (IRB). The DSMB will make recommendations concerning the continuation or conclusion of the study. The DSMB will monitor both safety and outcome data as part of the yearly review. Outcome evaluations will include review of data quality and timeliness, participant recruitment, accrual and retention, and review of interim "masked" outcome results on primary and secondary measures. Safety evaluations will include review of adverse events and symptom measures for each participant. The DSMB will further consider external factors such as scientific and therapeutic developments that may impact the safety or the ethics of the study. All serious adverse events will be reported to the members of the DSMB and the IRB according to CSMC IRB policies and procedures guidelines. A report of all non-serious adverse events will be provided to the DSMB yearly. PCORI will be informed of all actions taken by the IRB as part of the continuing review (see appendix D).

D. Emergency Procedures: Safety checks and suicide risk assessment will be performed at each point of contact and enrolled patients in both groups will be given an emergency phone number to contact the study staff in case of a psychiatric or medical emergency (given the FDA warning

of suicide risk following starting depression treatment). An on-site psychiatrist will provide emergency care for patients with increased suicide risk and suicidal behaviors. In-between visits; 24-hour emergency coverage is provided by a psychiatrist on-call for research subjects. All subjects are provided with the PI emergency contact number. Emergency care will be provided on-site for injuries arising due to the protocol only. If further treatment for a research-related injury is needed, it will be provided at no cost.

Section 9.0. Statistics

Sample Size and Power:

Aim 1: To compare the effectiveness of BA vs. MEDS, for depressed AHF patients.

Power Considerations: The main hypothesis to be tested is whether there is a difference in the change of PHQ-9 from baseline to 6 months between two groups (BA and MEDS).

The standard deviation for the two groups was estimated equal to 5.42 based on previous literature (Ekers, 2014) using the 95% confidence interval width asymptotic formulae for the difference between two change score means at 6 months, assuming the standard deviations were equal between groups. We assume a minimal detectable standardized mean difference (mean difference/pooled standard deviation) for PHQ-9 between arms (BA and MEDS) equal to 0.42 (Ekers, 2014) that corresponds to a minimal detectable mean difference of 2.27 in PHQ-9 score units if we use the estimated standard deviation. Under these assumptions, the required sample size for each group is 90 (Total = 180) patients using 5% significance, 80% power, and two-sided two sample equal-variance t-test. We argue that 2.27 PHQ-9 score units is a clinical meaningful difference because Richards (2016) uses 1.9 in PHQ-9 score units as equivalence margin for their non-inferiority trial between CBT and BA.

Table 1. Minimum detectable difference for secondary endpoints in Aim 1 with 80% power at 5% significance level

Endpoint	Standard Deviation	Minimum Detectable Difference
SF-12v2 (Physical)	3.28	1.4
SF-12v2 (Mental)	4.86	2
KCCQ (Quality of Life)	18.08	7.6
CBQ-HF (Emotional Well-being)	17.90	7.5

Table 1 presents the minimum detectable difference assuming a sample size of 90 patients for each group for the secondary endpoints SF-12v2, KCCQ and CBQ-HF with standard deviations estimated based on previous literature (Seerija, 2015 and Trivedi, 2016). For SF-12v2 and CBQ-HF, correlation between pre- and post- measures were assumed equal zero as a conservative estimate. For KCCQ, the largest 95% confidence interval width at 6 months was used similarly to PHQ-9.

Statistical Considerations: Descriptive measures followed by spaghetti plots will be presented. Multivariable Generalized Additive Model for Location, Scale and Shape (GAMLSS) (Rigby et al., 2005) will be fitted considering baseline response, time, treatment, and interaction between time and treatment as covariables with random effects describing repeated measures at 3, 6, and 12 months after treatment. In addition, admitting diagnosis, medical comorbidities, and other demographic, clinical, or utilization variables will be considered as control covariables. Time will be modeled as continuous variable using penalized cubic splines (Eilers et al., 1996) to avoid overfitting. Model diagnostics will be performed based on analysis of and Worm-plots (van Buuren et al., 2001). All tests of hypotheses will be two-sided with a significance level of 5%. Calculations will be performed using R, version 3.2.5 (R Core Team, 2016).

Aim 2: To compare the impact of BA vs. MEDS on Morbidity (as evidenced by ED visits, hospital readmissions, total days in the hospital), and Mortality among depressed AHF patients.

Power Considerations: The main hypothesis to be tested is whether there is difference for number of readmissions and ED visits. Baseline response rate and mean of exposure time are assumed equal to 1. Table 2 presents the minimum detectable response rate ratio (RR) using Poisson regression as function of R2 for regression of group variable on control variables with 80% of power at 5% significance (Freedland et al, 2016; Moraska et al., 2013).

Table 2. Minimum detectable response RR for Aim 2

R2	0.2	0.4	0.6	0.8
Response RR	1.57	1.68	1.88	2.43

Statistical Considerations: Recurring readmissions and ED visits times will be modeled with Mortality using Jointly Frailty Models (Rondeau et al., 2007) as a function of treatment. Smooth parameters will be estimated based on Shared Frailty models. The Length of Stay will be modeled as function of treatment using GAMLSS. Covariables as in Aim 1 will also be considered. All tests of hypotheses will be two-sided with a significance level of 5%. Calculations will be performed using R, version 3.2.5 (R Core Team, 2016).

Since we expanded our recruitment to the outpatient program, we would like to expand the sample size beyond the power calculation to 150 for each group (Total = 300 patients) to bring the study size close to equivalent heart failure studies and enable subgroup analyses. We estimate a dropout rate of 28%. Therefore, we plan on enrolling 416 patients (15% of to be approached eligible patients) in order to achieve a sample size of 300 patients for data analysis.

CONSORT Flow Diagram

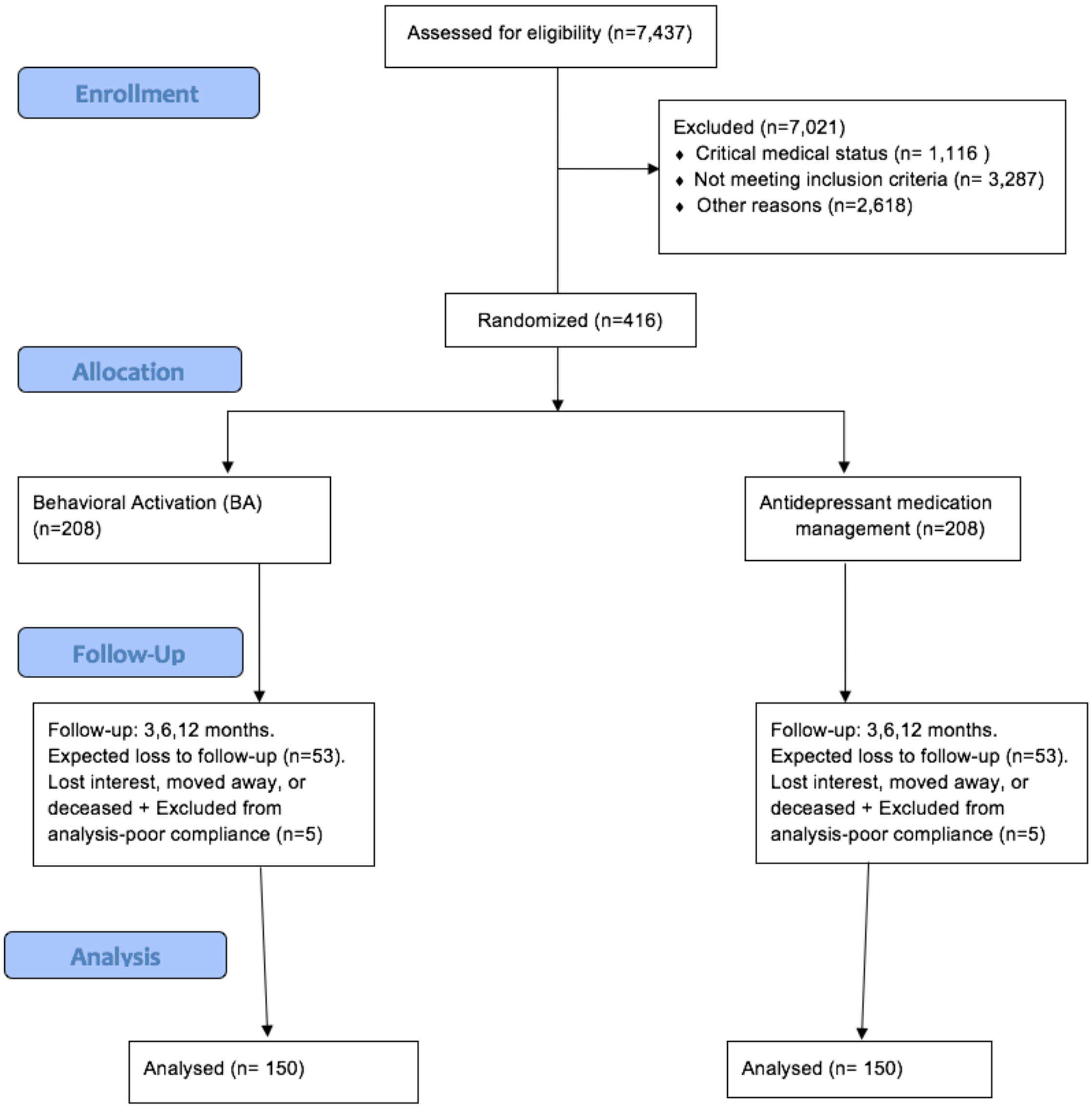

Analytic Plan

The primary outcome is Depressive symptom severity as measured by the PHQ-9 score (quantitative continuous variable) at 6 months. The secondary outcomes measured at 3, 6 and 12 months are: General physical and mental quality of life as measured by the SF-12v2 score (quantitative continuous variable), Heart failure-specific quality of life as measured by the KCCQ score (quantitative continuous variable), Caregiver burden as measured by the CBQ-HF score (quantitative continuous variable), Morbidity as measured by number of ED visits (quantitative discrete variable), readmissions (quantitative discrete variable), and total days in the hospital (quantitative discrete variable), and Mortality (qualitative nominal variable).

The covariables that will be considered are: Age, sex, race, ethnicity, marital status, employment, educational level, insurance, recruitment site (Inpatient or Outpatient), Ejection Fraction, NYHA Class, Medical History as detailed in the attached tables, Medications especially Angiotensin-converting enzyme inhibitor or angiotensin receptor blocker, β-Blocker, Loop Diuretic, Aldosterone antagonist, Opiate, and Antidepressant.

Aim 1: Summary statistics will be presented as percentages in the case of categorical variables and as means with standard deviations, medians with interquartile ranges in the case of continuous variables. Analysis of PHQ-9 as well as other outcomes questionnaires will be performed using multivariable Generalized Additive Model for Location, Scale and Shape (GAMLSS)[83] considering baseline response, time, treatment, and interaction between time and treatment as covariables with random effects describing repeated measures at 3, 6, 12 months after treatment. Time will be modeled as continuous variable using penalized cubic splines[84] to avoid overfitting. Random effects will also be added to address the correlation among patients at the same site.
Missing data will be treated as missing at random (MAR) using multivariate imputation by chained equations (van Buuren, 2007). The dataset will be imputed with values drawn from a distribution specifically modeled for each covariable, such that between 5 to 10 different imputed datasets will be created. Imputed values will be checked if are plausible as diagnostics. The outlined statistical analysis will be performed for each imputed dataset and results will be pooled according to Rubin (1987). In addition, a sensitivity analysis under the assumption of missing not at random (MNAR) will be performed using mixture models assuming various scenarios to assess the robustness of the results. In addition, covariables described above will be considered in the regression models. Model diagnostics will be performed based on analysis of residual plots and Worm-plots.

Aim 2: Analyses of recurring readmissions and ED visits times will be modeled with Mortality using Jointly Frailty Models as a function of treatment to model non-ignorable missing data generated by death. Time until death will be defined as the time between index time and the event. Time between recurring events such as ED visits and readmissions will be defined as the time between the last event and the next event. Individuals who are lost to follow-up are censored at the time of the last known contact. Smooth parameters will be estimated based on Shared Frailty models. The total days in the hospital will be modeled as function of treatment using Poisson regression and variations available in GAMLSS with random effects describing repeated measures.
In the HTE analysis, the presence of interaction between treatment and subgroup factor (Baseline PHQ, Heart Failure Type using Ejection Fraction, Heart Failure symptom severity using NYHA Classes) will be tested in the regression model (Wang et al., 2007). If the interaction is statistically significant, then the main analysis will be repeated for each group separately. Multiplicity will be addressed with Holm correction.
In the per-protocol analysis, propensity scores for adherence will be estimated as function of Percentage of completed BA sessions (BA arm) and Adapted Brief Medication Questionnaire (MEDS arm) at each time point and incorporated in the final models as inverse-probability weights.
All tests of hypotheses will be two-sided with a significance level of 5%. R version 3.2.5 will be used for calculations.

Section 10.0 Ethical considerations relating to the trial

Risk due to Study Procedures: Minimal psychological or emotional discomfort may arise due to the screening procedure and interviews /questionnaires conducted at each study visit. The instruments administered during the course of the study pose no specific risks or discomforts beyond those of a standard clinical interview situation such as feeling upset at a review of the psychiatric status or experiencing boredom or fatigue. There are questions that will be asked that may cause some subjects to feel uncomfortable. However, it will always be emphasized that all questions are optional, and participation is voluntary. Each subject will be screened for suicidal ideation and intent prior to study entry and any subject who is actively suicidal will be excluded from study participation and referred for appropriate care. The subject will be reminded that he/she may always decline to participate in this study and he/she may discontinue at any time.

Potential Risks: Behavioral activation will have minimal risk to the patients as study participation consists of education, learning, and completing a number of self-administered questionnaires and extracting data from a patient's medical chart. Patients using antidepressant may experience side effects from the medication, including bleeding risk, bradycardia, hypertension, electrocardiogram (EKG) changes, reduced cardiac conduction and output, arrhythmias, and sudden death. If patients experience these symptoms, they will be asked to stop using the antidepressant. To minimize the side effects, we will start with a low dose and work our way to the therapeutic dose with closed-monitoring for adverse effects mentioned above.
Adequacy of Protection against Risks: All patients meeting the eligibility criteria will be enrolled in the trial. The patient will receive an Informed Consent detailing the protocol, including research procedures and requirements for follow-up, as well as the risks and benefits of the study participation. The PI, Research Coordinator, and the research support team contact information will appear on the Informed Consent. Subjects will be informed that study participation is completely voluntary and that they can withdraw at any time. Once all data collection is complete, patient names will be replaced with a randomly-assigned study identification number for the purposes of de-identification. All identifiable data will be kept on a password protected file on a stationary desktop computer and kept separate from the patient medical record and other study data. The computer contains firewall and real-time anti-virus and anti-spyware protection that is kept up-to-date.

Section 11.0. Data Handling, Recordkeeping, and Quality Control and Assurance

Data and samples will be collected primarily using a computerized method (REDCap) in addition to the study Research Coordinator as supervised by the PI. All data and samples will be stored according to an assigned code number and the only personal identifiers associated with a sample include sex, age, and ethnicity. The code (with linkages) and any identifying data will be kept in a password-protected file located in a room separate from the other study files. The hard copy data files (documentation obtained from interviews, diagnostic procedures and testing) will be stored in the CSMC Psychiatry Research Offices. Digital files will be maintained by the

onsite data manager on a secured computer. Computer entries of data will be identified by code only. Additionally, a copy of the informed consent form and HIPPA consent form will be placed in the patient's medical chart and another copy will be given to the research coordinator. Informed consent forms will be stored in the Department of Psychiatry research office under lock and key. Data and samples will be collected primarily using a computerized method (REDCap) in addition to the study Research Coordinator as supervised by the PI. All data and samples will be stored according to an assigned code number and the only personal identifiers associated with a sample include sex, age, and ethnicity. The code (with linkages) and any identifying data will be kept in a password-protected file located in a room separate from the other study files. The hard copy data files (documentation obtained from interviews, diagnostic procedures and testing) will be stored in the CSMC Psychiatry Research Offices. Digital files will be maintained by the onsite data manager on a secured computer. Computer entries of data will be identified by code only. Data will be collected and recorded into a database system. Each record will include the subject's unique identifying number, demographic information, visit type, visit date, total scores and individual item scores on each of the measures. The Data Management Coordinator (study Research Coordinator) will review data integrity in terms of accuracy (using error alerts) and completion of all items on a daily basis. A record of the data will be printed for each visit and will be kept under double lock and key at the research data storage cabinet in the Psychiatric Research Division. An electronic backup copy of the data will be saved on the secure server upon data entry, in addition to weekly CD disks. A separate data copy on CD will be stored on a weekly basis offsite under double lock and key to enable data retrieval in the event of a catastrophe preventing access to the facility or the data. A backup CD will be created on a quarterly basis. The PI will oversee compliance with the procedures set forth. The database will be designed and managed by the Cedars-Sinai Biostatistics and Bioinformatics Research Center (BBRC)– a group that provides support for clinical trials throughout CSMC. Reports generated from the database will provide a basis for ongoing monitoring of subject accrual and retention, as well as completeness of data. These will be used to identify and resolve problems that may arise. Back-up copies of the database will be created daily per CSMC EIS standard protocol for all hospital-wide data servers. Access to data will be restricted to the study team but the code linking subject identification # to patient health information (PHI) will be restricted to only the PI and the study Research Coordinator. Data will only be released to a third party in an emergency medical situation or when required by law.

Section 12.0. Financing and Insurance

This study will be funded principally by PCORI. No costs to the participants.

Section 13.0. Publication Policy

Dissemination and Implementation Plan

The stakeholders and the investigator team will be facilitating the dissemination and implementation of the study results and their incorporation into practice. We will be enlisting the help of the leadership of diverse professional organizations from the HWA, patient partners, CSMC investigator team within the stakeholder team to help us disseminate the results of this study within their respective fields and to professional/provider audiences. Joint presentations will be conducted by our researchers and stakeholders in order to disseminate study results to patients, communities, and scientific conferences. Our patient partners will collaborate on

patient-centered materials including a website that details the results of the study. We will work with the HWP organizations (NAMI, The Life Adjustment, LifeREstyle, Painted Brain, and Clearview Treatment Team, Community Partnered-Participatory Research) and other Clinician Partners. Furthermore, the PI and co-investigators will be present the study findings at national professional cardiology and psychiatry conferences. Additionally, the study results will be made publicly available and accessible through the online portal of Barbra Streisand Women's Heart Center, in a sub-site on the Cedars-Sinai Center for Outcomes Research, which will explain characteristics, methodology and results of the study. Announcements of the study's progress will be distributed in lay language throughout the following CSMC newsletters geared towards patients and providers, including The Bridge, Cedars Science, and Cedars-Sinai Discoveries Magazine. In an effort to extend the dissemination of our findings to outside health care institutions, they will be shared with the NIH-funded Clinical Translational Science Institutes (CTSI) at UCLA and the American Heart Association. We will collaborate closely with the Cedars-Sinai communication team to communicate our study results and communicate implementation/educational efforts to other health systems. For example, the communications team operates Facebook, Instagram and Twitter accounts in an effort to enhance reader-friendliness. Along the duration of the study, participants will be emailed a satisfaction survey, and will be provided with sharing their comments, suggestions, and on way to improve the study. The results from the comparison arms may be documented into the Dissemination & Implementation Repository by PCORI to answer the following questions: Psychotherapy or Meds for Heart Failure Depression? Gaining control of your depressive symptoms during Heart Failure: Which depression treatment will work best for you? We will continue to collaborate with our stakeholders for identifying and addressing barriers to dissemination and implementation of the study results throughout the three years of the study. For example, the perspectives of the stakeholders are likely to differ widely, and at times will conflict. Conflicts will be resolved by consensus. With regard to dissemination, all stakeholders will be provided with data and information that are both meaningful and accessible and will be relevant to a given participant or stakeholder. After completion of the study, the results will be presented at weekly seminars hosted by the CSMC Heart Institute, open to the public, patients and their caregivers and NAMI general public weekly seminars.

Possible barriers to disseminating and implementing the results of this research

We designed our study to be generalizable to the community health setting (e.g., use of BA, low cost and burden to patient). The use of depression screening is common among outpatient and general practitioner settings. However, its use is limited in large hospitals and inpatient settings. Therefore, the identification of depressed HF patients at other inpatient settings may be a barrier of our protocol implementation. We will encourage the implementation of depression screening in outpatient and inpatient heart failure programs in order to improve detection and treatment initiation. Barriers to dissemination and implementation include: availability of providers to deliver therapy, comfort of primary care physicians or cardiologists in prescribing antidepressant medications, stigma around depression, difficulty for debilitated patients in attending additional appointments, complexity of patients and perceived importance of depression relative to other comorbidities. An additional barrier may be the generalizability of our setting-situated in a dense urban location albeit consisting of diverse ethnic and socioeconomic groups. The use of BA and its ease of remote delivery mode and low-cost personnel would address providers' availability to deliver this therapy. The study's antidepressant management guidelines and Care Manager role would enhance PMD and cardiologist comfort with administering MEDs. This study's dissemination and implementation

efforts include enhancing awareness of depressive symptoms in HF and enhancing the communication process between the provider and the patient's depressive symptoms, which could help address the stigma and deliver patient-centered interventions.

APPENDIX A: FLOWCHART OF PROCEDURES<br>Procedures	Baseline Visit	Visit #2 (Introductory Session)	3 months follow-up visit	6 months follow-up visit	12 months follow-up visit
Informed Consent	X				
MINI to confirm Diagnosis	X				
MOCA to ruled out cognitive disorder	X				
Randomization	X				
Primary outcome measure: PHQ-9	X		X	X	X
Secondary outcome measures: SF-12v2, KCCQ, CBQ-HF	X		X	X	X
Morbidity measures: ED visits, readmissions, total hospital days			X	X	X
Mortality rate			X	X	X
**Adapted Brief Medication Questionnaire**			X	X	X
Percentage of completed BA sessions			X	X	X
Behavioral Activation		X	Completed 12 weekly sessions with BA Therapist	Completed 3 monthly sessions with BA Therapist	Completed as needed contact
MEDS		X	Completed 12 weekly contacts with Care Manager	Completed 3 monthly contacts with Care Manager	Completed as needed contact

	Appendix B: Recommendations for Antidepressant Medications for HF Patients				
**Order**	**Medications**	**Side effects**<br><br>(rare/overdose cases) *	**Interacting Agents common to HF**	**Clinical Effect & Monitoring**	**Recommended Medication and dose**
1st line	SSRIs (sertraline, paroxetine, fluoxetine)	Sexual dysfunction, GI: decreased appetite, nausea, diarrhea, constipation, dry mouth. CNS: insomnia, sedation, agitation, tremors, headache, dizziness.	NSAIDS, oral anticoagulants, antiplatelets<br><br>Diuretics<br><br>Clonidine	Increased risk for bleeding<br><br>Hyponatremia<br><br>Hypothermia, sedation	Sertraline (50mg/day)
2	Dopaminergic and norepinephrine agents (bupropion)	Dry mouth, GI: constipation, nausea CNS: insomnia, dizziness, headache, agitation, anxiety. Weight loss, anorexia, myalgia, tremor, sweating, rash , hypertension	Clonidine<br><br>Warfarin	Hypothermia, sedation<br><br>Increased international normalized ratio	Recommended in HF patients with high BMI<br><br>OR hypotension
2	Noradrenergic and serotonergic agents (mirtazapine)	Flulike symptoms, changes in urinary symptoms, weight gain, GI: decreased appetite, nausea, diarrhea, constipation, dry mouth, CNS: sedation, confusion. Hypotension*	Clonidine<br><br>Warfarin	Hypothermia, sedation<br><br>Increased international normalized ratio	Recommended in HF patients with low BMI<br><br>OR Hypertension OR if patient is unresponsive to SSRIs or DNRIs
3	SNRI	Sexual	NSAIDS, oral	Increased risk	Use if patient

	(venlafaxine, duloxetine)	dysfunction, GI: decreased appetite, nausea, diarrhea, constipation, dry mouth. CNS: insomnia, sedation, headache. hyponatremia, hypertension	anticoagulants, antiplatelets	for bleeding	was unresponsive to SSRI, DNRI or mirtazapine
X	TCAs	Orthostatic hypotension, tachycardia, QT prolongation, weight gain			Not recommended for HF patients
X	MAOIs	Orthostatic hypotension, hypertension bradycardia, weight gain			Not recommended for HF patients
	1. Start medication according to guideline and conversation between primary care provider, patient and caregiver (12 weeks).				
	2. If side effects occur:<br>Consider drug-drug interactions:<br>a. serotonin syndrome: antidepressants, opioids, stimulants, 5-HT1 agonists, herbs, mood stabilizers, antipsychotics, antiemetics (ondansetron, metoclopramide), antibiotics (linezolid)<br>b. QTc Prolongation: SSRIs, antipsychotics, opioids, macrolides, fluoroquinolones, antiarrhythmics				
	3. Stop medication if side effects persist (10-14 days) after starting use, except sexual dysfunction				
	4. Refer to PI/Co-I in the event of:<br>a. refractory mood symptoms<br>b. development of psychotic and or manic symptoms<br>c. suicidal ideation<br>*following will be reported to the Data & Safety Monitoring Board (DSMB)<br>* PI/Co-I will be available after hours and patients will be given a 24-hour number to on-call Psychiatrist				

Appendix C: Patient/Caregiver-Reported and Clinician-Administered Instruments		
Name of instruments	How Administered?	Description
Patient Health Questionnaire (PHQ-9)	Self-administered	The PHQ-9[93] was developed as a self-report instrument that corresponded with the validated Primary Care Evaluation of Mental Disorders PRIME-MD clinician-administered instrument. Using data from nearly 4000 primary care outpatients, aged 18 or older, the PHQ-9 measures all nine dimensions of depression assessed in the DSM criteria for MDD on a 0-3 scale. Compared to a mental health professional's assessment, the PHQ-9 demonstrates a high degree of specificity and sensitivity (88% each). The PHQ has been extensively validated in outpatient medical patients, including primary care patients, and patients in specialty clinics with a variety of disorders
12-item short form (SF-12v2)	Self-administered	The SF-12v2 is a 12-item questionnaire used to assess generic health outcomes from the patient's perspective. Generic patient-reported outcome (PRO) measures like the SF-12v2 assess general physical and mental health-related quality of life (HRQoL), including the impact of any and all illnesses on a broad range of functional domains.
Kansas City Cardiomyopathy Questionnaire (KCCQ)	Self-administered	The KCCQ is a 23-item, self-administered instrument that quantifies physical function, symptoms (frequency, severity and recent change), social function, self-efficacy and knowledge, and HRQoL. In the KCCQ, an overall summary score can be derived from the physical function, symptom (frequency and severity), social function and HRQoL domains. For each domain, the validity, reproducibility, responsiveness and interpretability have been independently established. Scores are transformed to a range of 0-100, in which higher scores reflect better health status.
Caregiver Burden Questionnaire (CBQ-HF)	Self-administered	The Caregiver Burden Questionnaire - Heart Failure Version 3.0 (CBQ-HF) is a quantitative survey of 26 questions covering the past four weeks of the caregiver's experience is evaluated as caregiver burden. The questionnaire is based on interviews of HF caregivers and uses a 5-point Likert severity scale assessing 4 domains of physical, emotional/psychological, social and lifestyle burdens[81].
MINI International Neuropsychiatric Interview for the DSM-5 (MINI 7.02)	Clinician-administered	The MINI International Neuropsychiatric Interview for the DSM-5 (MINI 7.02) is a brief structured interview to check if the patient meets DSM-5 criteria for current Major Depressive Disorder, Persistent Depressive Disorder (Dysthymia), and Depressive Disorder Unspecified, and to rule out DSM-5 criteria for Bipolar, Psychotic, or Substance-induced Disorders.
The Montreal Cognitive Assessment (MoCA)	Clinician-administered	The Montreal Cognitive Assessment (MoCA).[64] The MoCA is a one-page test available online and is administered in approximately 10 minutes with maximum score of 30. It assesses short-term memory recall, visuospatial abilities, executive functions, attention, concentration, and working memory, language, and orientation to time and place.
Adapted Brief Medication Questionnaire	Clinician-administered	The Adapted Brief Medication Questionnaire will be used to assess antidepressant medication adherence in the MEDS arm patients after 3, 6, and 12 months of treatment. The scale is used to rank the degree of adherence instead of defining an absolute cutoff for adherence.

Appendix D: Reportable Adverse Events

Serious side effects associated with anti-depressants

- Common side effects associated with taking anti-depressants will not be reported (see appendix B). General common side effects include sedation and, decreased sexual desire, in addition to specific ones listed in Appendix B.
- Only serious side effects not anticipated in the typical adverse effect profiles of these medications will be reported. These side effects include serotonin syndrome and new onset suicidal ideations after starting antidepressant medications.

Severe suicidal ideation resulting in Suicidal attempts.

All reportable protocol deviations will be reported per Cedars Sinai Medical Center (CSMC) IRB policies and procedures.

All protocol deviations, Adverse Events, and Serious Adverse Events will be reported per Cedars Sinai Medical Center (CSMC) IRB policies and procedures. Additionally, only deaths specifically attributable to the study intervention will be reported to the IRB. For some context, the mortality rate for individuals with heart failure are as high as 25% at one year, with hospital readmission as high as 25%-50% within 6 months after discharge (Joynt, 2004). Therefore, a significant morbidity and mortality rate is expected for this population secondary to their medical illness irrespective to the effect of the study intervention and should not be considered a Serious Adverse Event for the purpose of IRB reporting. Patients with advanced heart failure are commonly admitted to the hospital for medical reasons, therefore for the purpose of reporting study-interventions-related events, any hospitalizations directly related to depressive symptoms, side effects of antidepressant medications or behavioral activation psychotherapy will be reported.

	Appendix B: Recommendations for Antidepressant Medications for HF Patients				
Order	Medications	Side effects (rare/overdose cases) *	Interacting Agents common to HF	Clinical Effect & Monitoring	Recommended Medication and dose
1st line	SSRIs (sertraline, paroxetine, fluoxetine)	Sexual dysfunction, GI: decreased appetite, nausea, diarrhea, constipation, dry mouth. CNS: insomnia, sedation, agitation, tremors, headache, dizziness.	NSAIDS, oral anticoagulants, antiplatelets<br><br>Diuretics<br><br>Clonidine	Increased risk for bleeding<br><br>Hyponatremia<br><br>Hypothermia, sedation	Sertraline (50mg/day)

2	Dopaminergic and norepinephrine agents (bupropion)	Dry mouth, GI: constipation, nausea CNS: insomnia, dizziness, headache, agitation, anxiety. Weight loss, anorexia, myalgia, tremor, sweating, rash , hypertension	Clonidine<br><br>Warfarin	Hypothermia, sedation<br><br>Increased international normalized ratio	Recommended in HF patients with high BMI<br><br>OR hypotension
2	Noradrenergic and serotonergic agents (mirtazapine)	Flulike symptoms, changes in urinary symptoms, weight gain, GI: decreased appetite, nausea, diarrhea, constipation, dry mouth, CNS: sedation, confusion. Hypotension*	Clonidine<br><br>Warfarin	Hypothermia, sedation<br><br>Increased international normalized ratio	Recommended in HF patients with low BMI<br><br>OR Hypertension OR if patient is unresponsive to SSRIs or DNRIs
3	SNRI (venlafaxine, duloxetine)	Sexual dysfunction, GI: decreased appetite, nausea, diarrhea, constipation, dry mouth. CNS: insomnia, sedation, headache. hyponatremia, hypertension	NSAIDS, oral anticoagulants, antiplatelets	Increased risk for bleeding	Use if patient was unresponsive to SSRI, DNRI or mirtazapine
X	TCAs	Orthostatic hypotension, tachycardia, QT prolongation, weight gain			Not recommended for HF patients
X	MAOIs	Orthostatic hypotension, hypertension bradycardia,			Not recommended for HF patients

		weight gain			
	1. Start medication according to guideline and conversation between primary care provider, patient and caregiver (12 weeks).				
	2. If side effects occur:<br>Consider drug-drug interactions:<br>a. serotonin syndrome: antidepressants, opioids, stimulants, 5-HT1 agonists, herbs, mood stabilizers, antipsychotics, antiemetics (ondansetron, metoclopramide), antibiotics (linezolid)<br>b. QTc Prolongation: SSRIs, antipsychotics, opioids, macrolides, fluoroquinolones, antiarrhythmics				
	3. Stop medication if side effects persist (10-14 days) after starting use, except sexual dysfunction				
	4. Refer to PI/Co-I in the event of:<br>a. refractory mood symptoms<br>b. development of psychotic and or manic symptoms<br>c. suicidal ideation<br>*following will be reported to the Data & Safety Monitoring Board (DSMB)<br>* PI/Co-I will be available after hours and patients will be given a 24-hour number to on-call Psychiatrist				

REFERENCES CITED

1. Association TCCotNYH. *Nomenclature and Criteria for Diagnosis of Diseases of the Heart and Great Vessels.* 9th ed. Boston, Mass:: Little, Brown & Co; 1994.
2. Sheehan DV, Sheehan KH, Shytle RD, et al. Reliability and validity of the Mini International Neuropsychiatric Interview for Children and Adolescents (MINI-KID). *The Journal of clinical psychiatry.* 2010;71(3):313-326.
3. (CDC) CfDCaP. Heart Failure Fact Sheet. 2016.
4. Bui AL, Horwich TB, Fonarow GC. Epidemiology and risk profile of heart failure. *Nature reviews Cardiology.* 2011;8(1):30-41.
5. (WHO) WHO. Depression and other common mental disorders: global health estimates. Accessed August, 2017.
6. Lane DA, Chong AY, Lip GY. Psychological interventions for depression in heart failure. *The Cochrane database of systematic reviews.* 2005(1):Cd003329.
7. Yohannes AM, Willgoss TG, Baldwin RC, Connolly MJ. Depression and anxiety in chronic heart failure and chronic obstructive pulmonary disease: prevalence, relevance, clinical implications and management principles. *International journal of geriatric psychiatry.* 2010;25(12):1209-1221.
8. Gottlieb SS, Khatta M, Friedmann E, et al. The influence of age, gender, and race on the prevalence of depression in heart failure patients. *Journal of the American College of Cardiology.* 2004;43(9):1542-1549.
9. Lichtman JH, Bigger JT, Jr., Blumenthal JA, et al. Depression and coronary heart disease: recommendations for screening, referral, and treatment: a science advisory from the American Heart Association Prevention Committee of the Council on Cardiovascular Nursing, Council on Clinical Cardiology, Council on Epidemiology and Prevention, and Interdisciplinary Council on

Quality of Care and Outcomes Research: endorsed by the American Psychiatric Association. *Circulation.* 2008;118(17):1768-1775.
10. Jeyanantham K, Kotecha D, Thanki D, Dekker R, Lane DA. Effects of cognitive behavioural therapy for depression in heart failure patients: a systematic review and meta-analysis. *Heart failure reviews.* 2017.
11. Abramson J, Berger A, Krumholz HM, Vaccarino V. Depression and risk of heart failure among older persons with isolated systolic hypertension. *Archives of internal medicine.* 2001;161(14):1725-1730.
12. Glassman AH, Shapiro PA. Depression and the course of coronary artery disease. *American Journal of Psychiatry.* 1998;155(1):4-11.
13. Cohen HW, Gibson G, Alderman MH. Excess risk of myocardial infarction in patients treated with antidepressant medications: Association with use of tricyclic agents. *American Journal of Medicine.* 2000;108(1):2-8.
14. Sherwood A, Blumenthal JA, Hinderliter AL, et al. Worsening depressive symptoms are associated with adverse clinical outcomes in patients with heart failure. *Journal of the American College of Cardiology.* 2011;57(4):418-423.
15. Turvey CL, Klein DM, Pies CJ, Arndt S. Attitudes about impairment and depression in elders suffering from chronic heart failure. *International journal of psychiatry in medicine.* 2003;33(2):117-132.
16. Johansson P, Dahlstrom U, Brostrom A. Consequences and predictors of depression in patients with chronic heart failure: implications for nursing care and future research. *Progress in cardiovascular nursing.* 2006;21(4):202-211.
17. Skotzko CE, Krichten C, Zietowski G, et al. Depression is common and precludes accurate assessment of functional status in elderly patients with congestive heart failure. *Journal of cardiac failure.* 2000;6(4):300-305.
18. Dracup K, Walden JA, Stevenson LW, Brecht ML. QUALITY-OF-LIFE IN PATIENTS WITH ADVANCED HEART-FAILURE. *Journal of Heart and Lung Transplantation.* 1992;11(2):273-279.
19. Mayou R, Blackwood R, Bryant B, Garnham J. CARDIAC-FAILURE - SYMPTOMS AND FUNCTIONAL STATUS. *Journal of Psychosomatic Research.* 1991;35(4-5):399-407.
20. Friedman MM, King KB. CORRELATES OF FATIGUE IN OLDER WOMEN WITH HEART-FAILURE. *Heart & Lung.* 1995;24(6):512-518.
21. Hawthorne MH, Hixon ME. Functional status, mood disturbance and quality of life in patients with heart failure. *Progress in cardiovascular nursing.* 1994;9(1):22-32.
22. Stewart AL, Greenfield S, Hays RD, et al. FUNCTIONAL STATUS AND WELL-BEING OF PATIENTS WITH CHRONIC CONDITIONS - RESULTS FROM THE MEDICAL OUTCOMES STUDY. *Jama-Journal of the American Medical Association.* 1989;262(7):907-913.
23. Blyth FM, Lazarus R, Ross D, Price M, Cheuk G, Leeder SR. Burden and outcomes of hospitalisation for congestive heart failure. *The Medical journal of Australia.* 1997;167(2):67-70.
24. Muller-Tasch T, Peters-Klimm F, Schellberg D, et al. Depression is a major determinant of quality of life in patients with chronic systolic heart failure in general practice. *Journal of cardiac failure.* 2007;13(10):818-824.
25. Sullivan M, Levy WC, Russo JE, Spertus JA. Depression and health status in patients with advanced heart failure: a prospective study in tertiary care. *Journal of cardiac failure.* 2004;10(5):390-396.
26. Friedman MM. Older adults' symptoms and their duration before hospitalization for heart failure. *Heart & Lung.* 1997;26(3):169-176.

27. Lesman-Leegte I, Jaarsma T, Sanderman R, Linssen G, van Veldhuisen DJ. Depressive symptoms are prominent among elderly hospitalised heart failure patients. *European journal of heart failure.* 2006;8(6):634-640.
28. Evangelista LS, Stromberg A, Dionne-Odom JN. An integrated review of interventions to improve psychological outcomes in caregivers of patients with heart failure. *Current opinion in supportive and palliative care.* 2016;10(1):24-31.
29. Dracup K, Evangelista LS, Doering L, Tullman D, Moser DK, Hamilton M. Emotional well-being in spouses of patients with advanced heart failure. *Heart & lung : the journal of critical care.* 2004;33(6):354-361.
30. Hooley PJ, Butler G, Howlett JG. The relationship of quality of life, depression, and caregiver burden in outpatients with congestive heart failure. *Congestive heart failure (Greenwich, Conn).* 2005;11(6):303-310.
31. Moraska AR, Chamberlain AM, Shah ND, et al. Depression, healthcare utilization, and death in heart failure: a community study. *Circulation Heart failure.* 2013;6(3):387-394.
32. Bhatt KN, Kalogeropoulos AP, Dunbar SB, Butler J, Georgiopoulou VV. Depression in heart failure: Can PHQ-9 help? *Int J Cardiol.* 2016;221:246-250.
33. Dekker Rl TE, Hoering LV, Bailey AL, Campbell CL, Wright JH, Bishop ML, Moser DK. A single cognitive behavioral therapy session improves short-term depressive symptoms in hospitalized patients with heart failure. *Circulation 130: A13961.* 2014.
34. Vaccarino V, Kasl SV, Abramson J, Krumholz HM. Depressive symptoms and risk of functional decline and death in patients with heart failure. *Journal of the American College of Cardiology.* 2001;38(1):199-205.
35. Diez-Quevedo C, Lupon J, Gonzalez B, et al. Depression, antidepressants, and long-term mortality in heart failure. *Int J Cardiol.* 2013;167(4):1217-1225.
36. Musselman DL, Marzec UM, Manatunga A, et al. Platelet reactivity in depressed patients treated with paroxetine - Preliminary findings. *Archives of General Psychiatry.* 2000;57(9):875-882.
37. Richards DA, Ekers D, McMillan D, et al. Cost and Outcome of Behavioural Activation versus Cognitive Behavioural Therapy for Depression (COBRA): a randomised, controlled, non-inferiority trial. *Lancet (London, England).* 2016;388(10047):871-880.
38. Fitzgerald DJ, Roy L, Catella F, Fitzgerald GA. PLATELET ACTIVATION IN UNSTABLE CORONARY-DISEASE. *New England Journal of Medicine.* 1986;315(16):983-989.
39. Ekers D, Webster L, Van Straten A, Cuijpers P, Richards D, Gilbody S. Behavioural activation for depression; an update of meta-analysis of effectiveness and sub group analysis. *PLoS One.* 2014;9(6):e100100.
40. LaghrissiThode F, Wagner WR, Pollock BG, Johnson PC, Finkel MS. Elevated platelet factor 4 and beta-thromboglobulin plasma levels in depressed patients with ischemic heart disease. *Biological psychiatry.* 1997;42(4):290-295.
41. Busch AM, Tooley EM, Dunsiger S, et al. Behavioral activation for smoking cessation and mood management following a cardiac event: results of a pilot randomized controlled trial. *BMC public health.* 2017;17(1):323.
42. Moore RC, Chattillion EA, Ceglowski J, et al. A randomized clinical trial of Behavioral Activation (BA) therapy for improving psychological and physical health in dementia caregivers: results of the Pleasant Events Program (PEP). *Behav Res Ther.* 2013;51(10):623-632.
43. Katon WJ. Epidemiology and treatment of depression in patients with chronic medical illness. *Dialogues Clin Neurosci.* 2011;13(1):7-23.

44. Unutzer J, Katon W, Sullivan M, Miranda J. Treating depressed older adults in primary care: Narrowing the gap between efficacy and effectiveness. *Milbank Quarterly.* 1999;77(2):225-+.
45. Unutzer J, Katon W, Williams JW, et al. Improving primary care for depression in late life - The design of a multicenter randomized trial. *Medical care.* 2001;39(8):785-799.
46. Unutzer J, Katon W, Callahan CM, et al. Collaborative care management of late-life depression in the primary care setting: a randomized controlled trial. *Jama.* 2002;288(22):2836-2845.
47. Celano CM, Healy B, Suarez L, et al. Cost-Effectiveness of a Collaborative Care Depression and Anxiety Treatment Program in Patients with Acute Cardiac Illness. *Value in health : the journal of the International Society for Pharmacoeconomics and Outcomes Research.* 2016;19(2):185-191.
48. Huffman JC, Mastromauro CA, Sowden G, Fricchione GL, Healy BC, Januzzi JL. Impact of a depression care management program for hospitalized cardiac patients. *Circulation Cardiovascular quality and outcomes.* 2011;4(2):198-205.
49. Rollman BL, Belnap BH, LeMenager MS, et al. Telephone-delivered collaborative care for treating post-CABG depression: a randomized controlled trial. *Jama.* 2009;302(19):2095-2103.
50. Lesperance F, Frasure-Smith N, Koszycki D, et al. Effects of citalopram and interpersonal psychotherapy on depression in patients with coronary artery disease: the Canadian Cardiac Randomized Evaluation of Antidepressant and Psychotherapy Efficacy (CREATE) trial. *Jama.* 2007;297(4):367-379.
51. Creber RM, Chen T, Wei C, Lee CS. Brief Report: Patient Activation among Urban Hospitalized Patients with Heart Failure. *Journal of cardiac failure.* 2017.
52. Wells KB, Jones L, Chung B, et al. Community-partnered cluster-randomized comparative effectiveness trial of community engagement and planning or resources for services to address depression disparities. *Journal of general internal medicine.* 2013;28(10):1268-1278.
53. Artinian NT, Artinian CG, Saunders MM. Identifying and treating depression in patients with heart failure. *The Journal of cardiovascular nursing.* 2004;19(6 Suppl):S47-56.
54. Guck TP, Elsasser GN, Kavan MG, Barone EJ. Depression and congestive heart failure. *Congestive heart failure (Greenwich, Conn).* 2003;9(3):163-169.
55. Rogers. *Diffusion of Innovations.* 4th ed. New York: The Free Press; 1995.
56. Taylor CB, Chang VY. Issues in the dissemination of cognitive-behavior therapy. *Nord J Psychiatry.* 2008;62 Suppl 47:37-44.
57. Unutzer J, Rubenstein L, Katon WJ, et al. Two-year effects of quality improvement programs on medication management for depression. *Archives of General Psychiatry.* 2001;58(10):935-942.
58. Saran RK, Puri A, Agarwal M. Depression and the heart. *Indian heart journal.* 2012;64(4):397-401.
59. Shapiro PA. Treatment of depression in patients with congestive heart failure. *Heart failure reviews.* 2009;14(1):7-12.
60. Cline CM, Israelsson BY, Willenheimer RB, Broms K, Erhardt LR. Cost effective management programme for heart failure reduces hospitalisation. *Heart (British Cardiac Society).* 1998;80(5):442-446.
61. Rutledge T, Reis VA, Linke SE, Greenberg BH, Mills PJ. Depression in heart failure a meta-analytic review of prevalence, intervention effects, and associations with clinical outcomes. *Journal of the American College of Cardiology.* 2006;48(8):1527-1537.

62. Parissis JT, Fountoulaki K, Paraskevaidis I, Kremastinos D. Depression in chronic heart failure: novel pathophysiological mechanisms and therapeutic approaches. *Expert Opin Investig Drugs.* 2005;14(5):567-577.
63. Ong MK, Romano PS, Edgington S, et al. Effectiveness of Remote Patient Monitoring After Discharge of Hospitalized Patients With Heart Failure: The Better Effectiveness After Transition -- Heart Failure (BEAT-HF) Randomized Clinical Trial. *JAMA Intern Med.* 2016;176(3):310-318.
64. Nasreddine ZS, Phillips NA, Bedirian V, et al. The Montreal Cognitive Assessment, MoCA: a brief screening tool for mild cognitive impairment. *Journal of the American Geriatrics Society.* 2005;53(4):695-699.
65. Carson N, Leach L, Murphy KJ. A re-examination of Montreal Cognitive Assessment (MoCA) cutoff scores. *International journal of geriatric psychiatry.* 2017.
66. Harris PA, Taylor R, Thielke R, Payne J, Gonzalez N, Conde JG. Research electronic data capture (REDCap)--a metadata-driven methodology and workflow process for providing translational research informatics support. *Journal of biomedical informatics.* 2009;42(2):377-381.
67. Shinohara K, Honyashiki M, Imai H, et al. Behavioural therapies versus other psychological therapies for depression. *The Cochrane database of systematic reviews.* 2013(10):Cd008696.
68. Dimidjian S, Barrera M, Jr., Martell C, Munoz RF, Lewinsohn PM. The origins and current status of behavioral activation treatments for depression. *Annual review of clinical psychology.* 2011;7:1-38.
69. Lejuez CW, Hopko DR, Acierno R, Daughters SB, Pagoto SL. Ten year revision of the brief behavioral activation treatment for depression: revised treatment manual. *Behavior modification.* 2011;35(2):111-161.
70. Huffman JC, DuBois CM, Healy BC, et al. Feasibility and utility of positive psychology exercises for suicidal inpatients. *General Hospital Psychiatry.* 2014;36(1):88-94.
71. Dham P, Colman S, Saperson K, et al. Collaborative Care for Psychiatric Disorders in Older Adults: A Systematic Review. *Canadian journal of psychiatry Revue canadienne de psychiatrie.* 2017:706743717720869.
72. Walker J, Hansen CH, Martin P, et al. Integrated collaborative care for major depression comorbid with a poor prognosis cancer (SMaRT Oncology-3): a multicentre randomised controlled trial in patients with lung cancer. *The Lancet Oncology.* 2014;15(10):1168-1176.
73. Rustad JK, Stern TA, Hebert KA, Musselman DL. Diagnosis and treatment of depression in patients with congestive heart failure: a review of the literature. *The primary care companion for CNS disorders.* 2013;15(4).
74. Erickson G, Hellerstein DJ. Behavioral activation therapy for remediating persistent social deficits in medication-responsive chronic depression. *Journal of psychiatric practice.* 2011;17(3):161-169.
75. Hellerstein DJ, Erickson G, Stewart JW, et al. Behavioral activation therapy for return to work in medication-responsive chronic depression with persistent psychosocial dysfunction. *Compr Psychiatry.* 2015;57:140-147.
77. Hopko DR, Lejuez CW, LePage JP, Hopko SD, McNeil DW. A brief behavioral activation treatment for depression - A randomized pilot trial within an inpatient psychiatric hospital. *Behavior modification.* 2003;27(4):458-469.

78. Green CP, Porter CB, Bresnahan DR, Spertus JA. Development and evaluation of the Kansas City Cardiomyopathy Questionnaire: a new health status measure for heart failure. *Journal of the American College of Cardiology.* 2000;35(5):1245-1255.
79. Humphrey L, Kulich K, Deschaseaux C, Blackburn S, Maguire L, Stromberg A. The Caregiver Burden Questionnaire for Heart Failure (CBQ-HF): face and content validity. *Health and quality of life outcomes.* 2013;11:84.
81. Riegel B, Lee CS, Dickson VV, Carlson B. An update on the self-care of heart failure index. *The Journal of cardiovascular nursing.* 2009;24(6):485-497.
82. Team RC. A language and environment for statistical computing. R Foundation for Statistical Computing, Vienna, Austria. 2016.
83. Rigby RA, Stasinopoulos DM. Generalized additive models for location, scale and shape. *Journal of the Royal Statistical Society: Series C (Applied Statistics).* 2005;54(3):507-554.
84. Eilers PH, Gampe J, Marx BD, Rau R. Modulation models for seasonal time series and incidence tables. *Stat Med.* 2008;27(17):3430-3441.
85. Ibrahim JG, Molenberghs G. Missing data methods in longitudinal studies: a review. *Test (Madrid, Spain).* 2009;18(1):1-43.
86. Rondeau V, Mathoulin-Pelissier S, Jacqmin-Gadda H, Brouste V, Soubeyran P. Joint frailty models for recurring events and death using maximum penalized likelihood estimation: application on cancer events. *Biostatistics (Oxford, England).* 2007;8(4):708-721.
87. Kahan BC, Jairath V, Dore CJ, Morris TP. The risks and rewards of covariate adjustment in randomized trials: an assessment of 12 outcomes from 8 studies. *Trials.* 2014;15:139.
88. van Buuren S, Fredriks M. Worm plot: a simple diagnostic device for modelling growth reference curves. *Stat Med.* 2001;20(8):1259-1277.
89. Beach J. Quality standards. *Community Pract.* 2013;86(11):3.
90. Kroenke K, Spitzer RL, Williams JBW. The PHQ-9 - Validity of a brief depression severity measure. *Journal of general internal medicine.* 2001;16(9):606-613.
91. Bozkurt Zincir S, Sunbul M, Zincir S, et al. Burden and depressive symptoms associated with adult-child caregiving for individuals with heart failure. *TheScientificWorldJournal.* 2014;2014:641817.
92. Kroenke K, Spitzer RL, Williams JB. The PHQ-9: validity of a brief depression severity measure. *Journal of general internal medicine.* 2001;16(9):606-613.
93. Cella D, Riley W, Stone A, et al. The Patient-Reported Outcomes Measurement Information System (PROMIS) developed and tested its first wave of adult self-reported health outcome item banks: 2005-2008. *Journal of clinical epidemiology.* 2010;63(11):1179-1194.
94. Radloff LS. THE CENTER FOR EPIDEMIOLOGIC STUDIES DEPRESSION SCALE A SELF REPORT DEPRESSION SCALE FOR RESEARCH IN THE GENERAL POPULATION. *Applied Psychological Measurement.* 1977;1(3):385-401.
95. Ustun TB, Chatterji S, Kostanjsek N, et al. Developing the World Health Organization Disability Assessment Schedule 2.0. *Bulletin of the World Health Organization.* 2010;88(11):815-823.

ADDITIONAL REFERENCES

- Abramson, J., Berger, A., Krumholz, H. M. & Vaccarino, V. (2001). Arch. Intern. Med. 161, 1725-1730.
- Acierno, R., Rheingold, A., Amstadter, A., Kurent, J., Amella, E., Resnick, H., Muzzy, W. & Lejuez, C. (2012). Am. J. Hosp. Palliat. Care 29, 13-25.

- Alosiami FD, Baker B. Clinical review of treatment options for major depressive disorder in patients with coronary heart disease. Saudi Med J. 2012 Nov;33(11):1159-68.
- Angermann CE, Gelbrich G, Störk S, et al., MOOD-HF Study Investigators and Committee Members, Effect of escitalopram on all-cause mortality and hospitalization in patients with heart failure and depression: the MOOD-HF randomized clinical trial, JAMA 315 (2016) 2683–2693.
- Artinian, N. T., Artinian, C. G. & Saunders, M. M. (2004). J. Cardiovasc. Nurs. 19, S47-56.
- Bashshur RL, Shannon GW, Smith BR, Alverson DC, Antoniotti N, Barsan WG, Bashshur N, Brown EM, Coye MJ, Doarn CR, Ferguson S, Grigsby J, Krupinski EA, Kvedar JC, Linkous J, Merrell RC, Nesbitt T, Poropatich R, Rheuban KS, Sanders JH, Watson AR, Weinstein RS, Yellowlees P. The empirical foundations of telemedicine interventions for chronic disease management. Telemed J E Health. 2014 Sep;20(9):769-800.
- Beach, S. R., Januzzi, J. L., Mastromauro, C. A., Healy, B. C., Beale, E. E., Celano, C. M. & Huffman, J. C. (2013). J. Psychosom. Res. 75, 409-413.
- Bekelman DB, Allen LA, McBryde CF, Hattler B, Fairclough DL, Havranek EP, Turvey C, Meek PM. Effect of a Collaborative Care Intervention vs Usual Care on Health Status of Patients With Chronic Heart Failure: The CASA Randomized Clinical Trial. JAMA Intern Med. 2018 Feb 26. doi:10.1001/jamainternmed.2017.8667.
- Bekelman DB, Havranek EP, Becker DM, Kutner JS, et al. Symptoms, depression, and quality of life in patients with heart failure. J Card Fail. 2007 Oct;13(8):643-8.
- Brouwers C, Christensen SB, Damen NL, Denollet J, et al. Antidepressant use and risk for mortality in 121,252 heart failure patients with or without a diagnosis of clinical depression. Int J Cardiol. 2016 Jan 15;203:867-73.
- Busch AM, Tooley EM, Dunsiger S, et al. Behavioral activation for smoking cessation and mood management following a cardiac event: results of a pilot randomized controlled trial. BMC public health. 2017;17(1):323.
- Cajanding RJ, 2016. The effectiveness of a nurse-led cognitive-behavioral therapy on the quality of life, self-esteem and mood among filipino patients living with heart failure: a randomized controlled trial. Appl. Nurs. Res. 31, 86–93.
- Celano CM, Healy B, Suarez L, et al. Cost-Effectiveness of a Collaborative Care Depression and Anxiety Treatment Program in Patients with Acute Cardiac Illness. Value in health : the journal of the International Society for Pharmacoeconomics and Outcomes Research. 2016;19(2):185-191
- Cleland (2015): Depression associated with 5-fold increased mortality risk in heart failure patients available at: https://www.escardio.org/The-ESC/Press-Office/Press-releases/Depression-associated-with-5-fold-increased-mortality-risk-in-heart-failure-pati Accessed on April 17, 2018.
- Cockayne S, Pattenden J, Worthy G, Richardson G, Lewin R. Nurse facilitated Self-management support for people with heart failure and their family carers (SEMAPHFOR): a randomised controlled trial. Int J Nurs Stud. 2014 Sep;51(9):1207-13.
- Cully JA, Stanley MA, Deswal A, Hanania NA, Phillips LL, Kunik ME. Cognitive-behavioral therapy for chronic cardiopulmonary conditions: preliminary outcomes from an open trial. Prim Care Companion J Clin Psychiatry. 2010;12(4). pii: PCC.09m00896.
- Dekker 2014a: Dekker RL, Tovar EG, Doering LV, Bailey AL, Campbell CL, Wright JH, Bishop ML, Moser DK (2014) A single cognitive behavior therapy session improves short-term depressive symptoms in hospitalized patients with heart failure. Circulation 130:A13961

- Dekker 2014b: Dekker RL. Patient perspectives about depressive symptoms in heart failure: a review of the qualitative literature. J Cardiovasc Nurs. 2014 Jan-Feb;29(1):E9-15.
- Dekker RL (2011) Cognitive therapy for depression in patients with heart failure: a critical review. Heart Fail Clin 7:127–141
- Dekker RL, Moser DK, Peden AR, Lennie TA (2012) Cognitive therapy improves three-month outcomes in hospitalized patients with heart failure. J Card Fail 18:19–20
- Delgado JF, Almenar L, González?Vilchez F, Arizón JM, Gómez M, Brossa V, Fernández J, Díaz B, Pascual D, Lage E, Sanz M. Health?related quality of life, social support, and caregiver burden between six and 120 months after heart transplantation. Clinical transplantation. 2015 Sep 1;29(9):771-80.
- Egede, L. E., Acierno, R., Knapp, R. G., Walker, R. J., Payne, E. H. & Frueh, B. C. (2016). J Clin Psychiatry 77, 1704-1711.
- Eilers, P. H., & Marx, B. D. (1996). Flexible smoothing with B-splines and penalties. Statistical science, 89-102.
- Ekers D, Webster L, Van Straten A, Cuijpers P, Richards D, Gilbody S. Behavioural activation for depression; an update of meta-analysis of effectiveness and sub group analysis. PLoS One. 2014;9(6):e100100.
- Ekers, D., Webster, L., Van Straten, A., Cuijpers, P., Richards, D. and Gilbody, S., 2014. Behavioural activation for depression; an update of meta-analysis of effectiveness and sub group analysis. PloS one, 9(6), p.e100100.
- Faller H, Steinbüchel T, Störk S, Schowalter M, Ertl G, Angermann CE. Impact of depression on quality of life assessment in heart failure. Int J Cardiol. 2010 Jul 9;142(2):133-7.
- Fisher L, Hessler D, Naranjo D, Polonsky W. AASAP: a program to increase recruitment and retention in clinical trials. Patient Educ Couns. 2012 Mar;86(3):372-7.
- Fraguas R, da Silva Telles RM, Alves TC, Andrei AM, Rays J, Iosifescu DV, et al. A double-blind, placebo-controlled treatment trial of citalopram for major depressive disorder in older patients with heart failure: the relevance of the placebo effect and psychological symptoms. Contemp Clin Trials 2009; 30:205-11.
- Freedland KE, Carney RM, Rich MW, Steinmeyer BC, Rubin EH (2015) Cognitive behavior therapy for depression and self-care in heart failure patients: a randomized clinical trial. JAMA Intern Med 175:1773–1782
- Freedland KE, Carney RM, Rich MW, Steinmeyer BC, Skala JA, Dávila-Román VG. Depression and Multiple Rehospitalizations in Patients With Heart Failure. Clin Cardiol. 2016 May;39(5):257-62.
- Friedman MB, Furst L, William KA. Today's Geriatric Medicine. 2013. Vol. 6 No. 5 P. 24. Accessed on March 25, 2018 at http://www.todaysgeriatricmedicine.com/archive/090913p24.shtml
- Gary RA, Dunbar SB, Higgins MK, Musselman DL, Smith AL (2010) Combined exercise and cognitive behavioral therapy improves outcomes in patients with heart failure. J Psychosom Res 69:119–131
- Gottlieb SS, Khatta M, Friedmann E, et al. The influence of age, gender, and race on the prevalence of depression in heart failure patients. Journal of the American College of Cardiology. 2004;43(9):1542-1549.
- Gottlieb SS, Kop WJ, Thomas SA, Katzen S, Vesely MR, Greenberg N, et al. A double-blind placebo-controlled pilot study of controlled-release paroxetine on depression and quality

of life in chronic heart failure.Am Heart J 2007;153:868-73 Guck, T. P., Elsasser, G. N., Kavan, M. G. & Barone, E. J. (2003). Congest. Heart Fail. 9, 163-169.

- Hanyu N, Toy W, Burgess D, Wise K, Nauman DJ, Crispell K, Hershberger RE. Comparative responsiveness of Short-Form 12 and Minnesota Living with Heart Failure Questionnaire in patients with heart failure. Journal of Cardiac Failure. 2000 Jun 1;6(2):83-91.
- Hopko, DR., Lejuez, CW., LePage, JP., Hopko, SD. & McNeil, DW. (2003). Behav. Modif. 27, 458-469.
- Huffman JC, Mastromauro CA, Sowden G, Fricchione GL, Healy BC, Januzzi JL. Impact of a depression care management program for hospitalized cardiac patients. Circulation Cardiovascular quality and outcomes. 2011;4(2):198-205.
- IsHak WW, Steiner AJ, Klimowicz A, Kauzor K, Dang J, Vanle B, Elzahaby C, Reid M, Sumner L, Danovitch I. Major Depression Comorbid with Medical Conditions: Analysis of Quality of Life, Functioning, and Depressive Symptom Severity. Psychopharmacol Bull. 2018 Jan 15;48(1):8-25.
- Jeyanantham K, Kotecha D, Thanki D, Dekker R, Lane DA. Effects of cognitive behavioural therapy for depression in heart failure patients: a systematic review and meta-analysis. Heart Fail Rev. 2017 Nov;22(6):731-741.
- Joynt K, Whellan D, Connor C. Why is Depression Bad for the Failing Heart? A Review of the Mechanistic Relationship Between Depression and Heart Failure. Journal of Cardiac Failure. 2004;10(3):258-267.
- Katon W, Von Korff M, Lin E, Simon G, Walker E, Bush T, Ludman E. Collaborative management to achieve depression treatment guidelines. J Clin Psychiatry. 1997;58 Suppl 1:20-3.
- Katon WJ. Epidemiology and treatment of depression in patients with chronic medical illness. Dialogues Clin Neurosci. 2011;13(1):7-23.
- Klinkman, M. S. (1997). Gen. Hosp. Psychiatry 19, 98-111.
- Mårtensson J, Dracup K, Canary C, Fridlund B. Living with heart failure: depression and quality of life in patients and spouses. J Heart Lung Transplant. 2003 Apr;22(4):460-7.
- Mavrides N, Nemeroff C. Treatment of depression in cardiovascular disease. Depress Anxiety. 2013 Apr;30(4):328-41.
- McMurray JJ, Adamopoulos S, Anker SD, et al. ESC Committee for Practice Guidelines. ESC Guidelines for the diagnosis and treatment of acute and chronic heart failure 2012: The Task Force for the Diagnosis and Treatment of Acute and Chronic Heart Failure 2012 of the European Society of Cardiology. Developed in collaboration with the Heart Failure Association (HFA) of the ESC. Eur Heart J 2012;33:1787–847.
- Mohr, D. C., Hart, S. L., Julian, L., Catledge, C., Honos-Webb, L., Vella, L. & Tasch, E. T. (2005). Arch. Gen. Psychiatry 62, 1007-1014.
- Mohr, D. C., Ho, J., Duffecy, J., Reifler, D., Sokol, L., Burns, M. N., Jin, L. & Siddique, J. (2012). JAMA 307, 2278-2285.
- Mohr, D. C., Likosky, W., Bertagnolli, A., Goodkin, D. E., Van Der Wende, J., Dwyer, P. & Dick, L. P. (2000). J. Consult. Clin. Psychol. 68, 356-361.
- Moore RC, Chattillion EA, Ceglowski J, et al. A randomized clinical trial of Behavioral Activation (BA) therapy for improving psychological and physical health in dementia caregivers: results of the Pleasant Events Program (PEP). Behav Res Ther. 2013;51(10):623-632.
- Newhouse, A. & Jiang, W. (2014). Heart Fail. Clin. 10, 295-304.

- Nicholas Dionne-Odom J, Hooker SA, Bekelman D, Ejem D, McGhan G, Kitko L, Strömberg A, Wells R, Astin M, Metin ZG, Mancarella G, Pamboukian SV, Evangelista L, Buck HG, Bakitas MA; IMPACT-HF National Workgroup. Family caregiving for persons with heart failure at the intersection of heart failure and palliative care: a state-of-the-science review. Heart Fail Rev. 2017 Sep;22(5):543-557.
- Ong MK, Romano PS, Edgington S, Aronow HU, Auerbach AD, Black JT, De Marco T, Escarce JJ, Evangelista LS, Hanna B, Ganiats TG, Greenberg BH, Greenfield S, Kaplan SH, Kimchi A, Liu H, Lombardo D, Mangione CM, Sadeghi B, Sadeghi B,Sarrafzadeh M, Tong K, Fonarow GC; Better Effectiveness After Transition–Heart Failure (BEAT-HF) Research Group. Effectiveness of Remote Patient Monitoring After Discharge of Hospitalized Patients With Heart Failure: The Better Effectiveness After Transition -- Heart Failure (BEAT-HF) Randomized Clinical Trial. JAMA Intern Med. 2016 Mar;176(3):310-8. doi:10.1001/jamainternmed.2015.7712. Erratum in: JAMA Intern Med. 2016 Apr;176(4):568.
- Patrick-Lake B. Patient engagement in clinical trials: The Clinical Trials Transformation Initiative's leadership from theory to practical implementation. Clin Trials. 2018 Feb;15(1_suppl):19-22.
- Polenick, C. A. & Flora, S. R. (2013). The Behavior analyst 36, 35-55.
- Psotka MA, von Maltzahn R, Anatchkova M, Agodoa I, Chau D, Malik FI, Patrick DL, Spertus JA, Wiklund I, Teerlink JR. Patient-Reported Outcomes in Chronic Heart Failure: Applicability for Regulatory Approval. JACC Heart Fail. 2016 Oct;4(10):791-804.
- Quijano, L. M., Stanley, M. A., Petersen, N. J., Casado, B. L., Steinberg, E. H., Cully, J. A. & Wilson, N. L. (2007). J. Appl. Gerontol. 26, 139-156.
- R Core Team (2016). A language and environment for statistical computing. R Foundation for Statistical Computing, Vienna, Austria.
- Rajeswaran T, Plymen CM, Doherty AM. The effect of antidepressant medications in the management of heart failure on outcomes: mortality, cardiovascular function and depression - a systematic review. Int J Psychiatry Clin Pract. 2017 Nov 26:1-6.
- Richards DA, Ekers D, McMillan D, et al. Cost and Outcome of Behavioural Activation versus Cognitive Behavioural Therapy for Depression (COBRA): a randomised, controlled, non-inferiority trial. Lancet (London, England). 2016;388(10047):871-880.
- Richards DA, Ekers D, McMillan D, et al. Cost and Outcome of Behavioural Activation versus Cognitive Behavioural Therapy for Depression (COBRA): a randomised, controlled, non-inferiority trial. Lancet (London, England). 2016;388(10047):871-880.
- Rigby RA, Stasinopoulos DM. (2005) Generalized additive models for location, scale and shape. Journal of the Royal Statistical Society: Series C (Applied Statistics). 54(3):507-54.
- Rollman BL, Belnap BH, LeMenager MS, et al. Telephone-delivered collaborative care for treating post-CABG depression: a randomized controlled trial. JAMA. 2009;302(19):2095-2103.
- Rondeau, Virginie, Simone Mathoulin-Pelissier, Hélène Jacqmin-Gadda, Véronique Brouste, and Pierre Soubeyran. "Joint frailty models for recurring events and death using maximum penalized likelihood estimation: application on cancer events." Biostatistics, no. 4 (2007): 708-721.
- Rubin DB. Multiple Imputation for Nonresponse in Surveys. New York: John Wiley & Sons; 1987.

- Rustad JK, Stern TA, Hebert KA, Musselman DL. Diagnosis and treatment of depression in patients with congestive heart failure: a review of the literature. Prim Care Companion CNS Disord. 2013;15(4).
- Shudo Y, Yamamoto T. Assessing the relationship between quality of life and behavioral activation using the Japanese Behavioral Activation for Depression Scale-Short Form. PLoS One. 2017 Sep 28;12(9):e0185221
- Sokoreli I, Pauws SC, Steyerberg EW, de Vries GJ, Riistama JM, Tesanovic A, Kazmi S, Pellicori P, Cleland JG, Clark AL. Prognostic value of psychosocial factors for first and recurrent hospitalizations and mortality in heart failure patients: insights from the OPERA-HF study. Eur J Heart Fail. 2018 Apr;20(4):689-696.
- Sreeja MS, Dharan D. Assessment of Care Giver Burden Among Patients With Heart Failure In A Tertiary Care Hospital. Int J Recent Sci Res. 2015;6(9), 6405-6408.
- Trivedi R, Slightam C, Fan VS, Rosland AM, Nelson K, Timko C, Asch SM, Zeliadt SB, Heidenreich P, Hebert PL, Piette JD. A couples' based self-management program for heart failure: results of a feasibility study. Frontiers in public health. 2016 Aug 29;4:171.
- Trombello JM, South C, Cecil A, Sánchez KE, Sánchez AC, Eidelman SL, Mayes TL, Kahalnik F, Tovian C, Kennard BD, Trivedi MH. Efficacy of a Behavioral Activation Teletherapy Intervention to Treat Depression and Anxiety in Primary Care VitalSign6 Program. Prim Care Companion CNS Disord. 2017 Oct 19;19(5). pii:17m02146.
- Unutzer J, Katon W, Callahan CM, et al. Collaborative care management of late-life depression in the primary care setting: a randomized controlled trial. Jama. 2002;288(22):2836-2845.
- Unutzer J, Katon W, Sullivan M, Miranda J. Treating depressed older adults in primary care: Narrowing the gap between efficacy and effectiveness. Milbank Quarterly. 1999;77(2):225.
- Unutzer J, Katon W, Williams JW, et al. Improving primary care for depression in late life - The design of a multicenter randomized trial. Medical care. 2001;39(8):785-799.
- Vaccarino V, Kasl SV, Abramson J, Krumholz HM. Depressive symptoms and risk of functional decline and death in patients with heart failure. J Am Coll Cardiol. 2001 Jul;38(1):199-205.
- van Buuren and Fredriks M. (2001) Worm plot: simple diagnostic device for modelling growth reference curves. Statistics in Medicine, 20, 1259–1277.
- van Buuren S. Multiple imputation of discrete and continuous data by fully conditional specification. Stat Methods Med Res. 2007 Jun;16(3):219-42.
- Wang, R., Lagakos, S.W., Ware, J.H., Hunter, D.J. and Drazen, J.M., 2007. Statistics in medicine—reporting of subgroup analyses in clinical trials. New England Journal of Medicine, 357(21), pp.2189-2194.
- Woltz PC, Chapa DW, Friedmann E, Son H, Akintade B, Thomas SA. Effects of interventions on depression in heart failure: a systematic review. Heart Lung 2012 Sep-Oct;41(5):469-83.
- Xiong GL, Fiuzat M, Kuchibhatla M, et al., SADHART-CHF Investigators, Health status and depression remission in patients with chronic heart failure: patient-reported outcomes from the SADHARTCHF trial, Circ. Heart Fail. 5 (2012) 688–692.
- Zweben A, Fucito LM, O'Malley SS. Effective Strategies for Maintaining Research Participation in Clinical Trials. Drug Inf J. 2009 Jul;43(4).
